# Transcriptome and metabolome analysis reveals PRV XJ delgE/gI/TK protects intracranially infected mice from death by regulating the inflammation

**DOI:** 10.3389/fmicb.2024.1374646

**Published:** 2024-03-14

**Authors:** Lei Xu, Yang Zhang, Qian Tao, Tong Xu, Feng-qin Lee, Li-shuang Deng, Zhijie Jian, Jun Zhao, Yanting Yang, Siyuan Lai, Yuan-cheng Zhou, Zhi-wen Xu, Ling Zhu

**Affiliations:** ^1^Key Laboratory of Animal Diseases and Human Health of Sichuan Province, College of Veterinary Medicine, Sichuan Agricultural University, Chengdu, China; ^2^Livestock and Poultry Biological Products Key Laboratory of Sichuan Province, Sichuan Animal Science Academy, Chengdu, China; ^3^Animal Breeding and Genetics Key Laboratory of Sichuan Province, Sichuan Animal Science Academy, Chengdu, China

**Keywords:** pseudorabies virus, inflammation, intracranial infection, transcriptome, metabolome

## Abstract

Pseudorabies virus can cause inflammation in the central nervous system and neurological symptoms. To further investigate the protective mechanism of PRV XJ delgE/gI/TK in the central nervous system, an intracranial PRV-infection mice model was developed. The results demonstrated that immunization with PRV XJ delgE/gI/TK successfully prevented death caused by PRV-intracranial infection. Subsequently, the brains were collected for transcriptome and metabolome analysis. GO and KEGG enrichment analysis indicated that the differentially expressed genes were primarily enriched in pathways such as TNF, NOD-like receptor, JAK–STAT, MAPK, IL-17 and apoptosis signaling. Metabolomics analysis revealed that the differential metabolites were mainly associated with pathways such as fatty acid degradation, arachidonic acid metabolism, linoleic acid metabolism and unsaturated fatty acid biosynthesis. The combined analysis of metabolites and differentially expressed genes revealed a strong correlation between the differential metabolites and TNF, PI3K, and MAPK signaling pathways. Anti-inflammatory metabolites have been shown to inhibit the inflammatory response and prevent mouse death caused by PRV infection. Notably, when glutathione was injected intracranially and dihydroartemisinin was injected intraperitoneally, complete protection against PRV-induced death in mice was observed. Moreover, PRV activates the PI3K/AKT signaling pathway. In conclusion, our study demonstrates that PRV XJ delgE/gI/TK can protects intracranially infected mice from death by regulating various metabolites with anti-inflammatory functions post-immunization.

## 1 Introduction

Pseudorabies (PR), also known as Aujeszky’s disease, was first reported in the United States in 1813 and has since spread worldwide, affecting a wide range of hosts. Most infected animals exhibit symptoms such as fever, itching, and encephalomyelitis (Zheng et al., 2022). The symptoms of pseudorabies virus (PRV) infection vary depending on the stage of infection in pigs. Newborn piglets experience diarrhea, fever, ataxia, convulsions, coma, and death (Ren et al., 2023). Weaned piglets display neurological symptoms, vomiting, and diarrhea. Nursery pigs primarily exhibit fever, cough, decreased feed intake, and respiratory difficulties. In early pregnancy, miscarriages occur approximately 20 days after infection, while late gestation often results in stillbirths and mummifications. Breeding pigs may experience sterility. According to the 2019 to 2021 OIE report, PRV is currently found mainly in domestic pigs in Argentina, Bosnia, China, Croatia, Cuba, France, Hungary, Italy, Mexico, Papua New Guinea, Poland, Portugal, Spain and the United States of America. Through effective vaccination and eradication measures, several countries including Germany, the United Kingdom, Ireland, South Korea, Sweden, Colombia, Denmark, and New Zealand have successfully eliminated PRV in domestic pigs (Liu et al., 2022). However, maintaining this elimination has proven to be challenging, as demonstrated by second outbreaks in Argentina in 2019, and France and Mexico in 2020.

PRV is a herpes A virus that primarily infects the peripheral nervous system. It can infect a wide range of animals, such as pigs, ruminants (sheep and cows), carnivores (minks and foxes), and rodents. However, pigs, including wild boars, are the natural host for this pathogen (Xiong et al., 2022). Interestingly, in these non-natural hosts, PRV always causes a severe acute and lethal neuropathy known as the ‘mad itch,’ which is uncommon in swine (Laval and Enquist, 2020). This results in high fever, increased intracranial pressure, cerebral edema, and eventually coma or death. Pathological manifestations include vacuolar degeneration, neuronophagy, and inflammatory cell infiltration (Xu et al., 2022). With the exception of pigs, almost all mammals infected with PRV develop severe neurological symptoms and die. Although rare, there have been reports of PRV infecting humans. Previous studies have detected PRV antibodies in cases of immunocompetent human encephalitis, but the virus itself was not isolated (Wang et al., 2022). Qingyun Liu reported the first isolation of a human PRV strain from the cerebrospinal fluid of encephalitis patients, confirming that PRV infection in humans can cause severe encephalitis. Early symptoms include headache, memory loss, seizures, and disturbances in consciousness. Despite antiviral treatment, permanent eye damage, blindness, mild memory impairment, minimally conscious state, and persistent vegetative status were observed as clinical outcomes (Liu et al., 2021). Yang et al. (2019) found that individuals rescued from PRV infection exhibited severe sequelae of central nervous system damage, such as delayed response and occasional seizures. Recent reports on human PRV infection cases have highlighted the significant threat that PRV poses to public health (Ou et al., 2020).

Long-chain unsaturated fatty acids primarily serve as sources of energy and membrane components. They can also be converted into bioactive lipid metabolites by symbiotic bacteria, resulting in the production of various fatty acid species, including linoleic acid and conjugated linoleic acid. These metabolites are involved in regulating immune responses (Peng and Biswas, 2017). Linoleic acid, α-linolenic acid, and conjugated linoleic acid have been shown to possess numerous health benefits, such as antiviral, anticancer, anti-inflammatory, and antioxidant properties (Maggiora et al., 2004; O'Shea et al., 2012). Conjugated linoleic acid can enhance antiviral immunity by increasing the population of porcine CD8 α/β (positive) lymphocytes (Bassaganya-Riera et al., 2001). Furthermore, 3’-Deoxy-3′,4′-didehydro-cytidine (ddhC) is the sole known free base of the human antiviral small molecule DDHC-triphosphate (ddhCTP) (Mehta et al., 2022). In conclusion, the host possesses a variety of antiviral small molecules or metabolites that exhibit antiviral, anti-inflammatory, and antioxidant functions following pathogen infection.

Given the current global epidemic of PRV variant strains in pigs, PRV delgE/gI/TK strains have been demonstrated to effectively protect pigs from PRV epidemic strain infection. Previous studies have reported that PRV delgE/gI/TK strains can prevent death in mice caused by PRV variant strain infection. However, they are unable to prevent PRV variant strains from colonizing the central nervous system. This is due to the lack of the TK gene in PRV delgE/gI/TK strains, which prevents their entry into the central nervous system and activation of immune cells such as astrocytes, microglia, and oligodendrocytes, thereby protecting the central nervous system (Pomeranz et al., 2005; Klein et al., 2017). The mechanism by which PRV delgE/gI/TK strain affects the clearance of virus in the central nervous system and the protection of the central nervous system is still unclear. Therefore, it is necessary to understand the protective effect of PRV delgE/gI/TK on the central nervous system. In this study, a cranial infection model of mice with PRV was used to screen cranial differential metabolites and differential expression genes among immunization-challenged group, challenged group, and mock group. The aim was to explore the mechanism by which PRV delgE/gI/TK regulates intracranial genes or metabolites to protect the central nervous system. This research provides a scientific and theoretical basis for the development of anti-PRV drugs.

## 2 Materials and methods

### 2.1 Virus and cells

PRV XJ virus was isolated, identified, and stored in our laboratory. C57BL/6 J mice were purchased from Beijing Huafukang Biotechnology Co., LTD. The mice were kept at room temperature (23 ± 1.5°C) with free access to food and water.

### 2.2 Determination of LD_50_ in intracranial infection mouse model

The PRV XJ strain was diluted 10-fold (10^–1.0^−10^–7.0^) with serum-free DMEM. A total of forty-eight 8-week-old male C57BL/6 J mice were randomly divided into 7 experimental groups (groups A-G) and a mock group (group H), with 6 mice in each group. The C57BL/6 J mice were anesthetized by intraperitoneal injection of 1% sodium pentobarbital. A sagittal incision (1.0–1.5 cm) was made on the scalp to expose the calvarium. A small parietal hole was created on the sagittal suture of the skull. The microinjector was then positioned at the right caudate nucleus (1 mm forward and 2 mm right of the anterior fontanelle) and vertically punctured 3 mm. The PRV XJ strain diluent (20 μL) was injected into all mice in groups A-G at a rate of 2 μL/min through the microinjector. The mock group (group H) was injected with the same dose of DMEM. The morbidity and death of mice were observed and recorded daily for approximately 7 days after inoculation. And LD_50_ was calculated using the Reed-Muench method.

### 2.3 Construction of mouse infection model

Fifteen C57BL/6 J mice were randomly divided into 3 groups, with 5 mice in each group. Group A was the immunization-challenged group, group B was the challenged group, and group C was the mock group. In group A, mice were immunized intramuscularly with 0.2 mL of 10^6^ TCID_50_ PRV XJ delgE/gI/TK. Two weeks after the initial immunization, a booster immunization with the same dose of PRV XJ delgE/gI/TK was administered. Group B and Group C were immunized intramuscularly with the same dose of serum-free DMEM.

According to the results of viral LD_50_ determination, at the fourth week after the first immunization, group A and group B mice were, respectively, treated with 20 μL of 10*LD_50_ PRV XJ strain in the intracranial cavity. Mice in group C were injected with 20 μL DMEM in the intracranial cavity. The clinical symptoms and death of mice were observed daily. Tissue and serum samples were collected from the dying or dead mice, including lungs, brains, livers, kidneys, spleens, and intestines. The samples for omics sequencing and validation experiments were stored at −80°C, and the samples for preparation of histopathological sections were fixed with 4% paraformaldehyde. When all intracranial challenge mice died, all mice in the mock group and immunization-challenged groups were humanely euthanized with an overdose of pentobarbitalum natricum (40 mg/kg). Samples were collected and stored as described above.

### 2.4 The effect of metabolites on PRV intracranial infection mice

Sixty C57BL/6 J mice were randomly divided into 10 groups, each containing 6 mice: histidine intraperitoneal injection group (histidine i.p), glutathione intraperitoneal injection group (glutathione i.p), tyrosine intraperitoneal injection group (tyrosine i.p), dihydroartemisinin intraperitoneal injection group (dihydroartemisinin i.p), histidine intracranial injection group (histidine i.c), glutathione intracranial injection group (glutathione i.c), tyrosine intracranial injection group (tyrosine i.c), dihydroartemisinin intracranial injection group (dihydroartemisinin i.c), the challenged group, and mock group. All the metabolites treatment groups and the challenged group received intracranial injection of 20 μL 10*LD_50_ PRV XJ strain. The mock group received intracranial injection with the same 20 μL DMEM. After 48 h, each metabolites treatment group was separately treated. The histidine i.p group was intraperitoneally injected with 0.2 mL of histidine (15 mg/mL), the glutathione i.p group was intraperitoneally injected with 0.2 mL of glutathione (15 mg/mL), the tyrosine i.p group was intraperitoneally injected with 0.2 mL of tyrosine (10 mg/mL), and the dihydroartemisinin i.p group was intraperitoneally injected with 0.2 mL of dihydroartemisinin (1 mg/mL). The histidine i.c group was intracranially injected with 20 μL of histidine (15 mg/mL), the glutathione i.c group was intracranially injected with 20 μL of glutathione (15 mg/mL), the tyrosine i.c group was intracranially injected with 20 μL of tyrosine (1 mg/mL), and the dihydroartemisinin i.c group was intracranially injected with 20 μL of dihydroartemisinin (1 mg/mL). The death of mice was recorded daily, and clinical symptoms were observed and scored according to Supplementary Table 1. Tissue and serum samples were collected from the dying or dead mice during the experiment, including lungs, brains, livers, kidneys, spleens, and intestines. On day 7, all mice were sacrificed using excess pentobarbital sodium. All the samples were stored at −80°C.

### 2.5 Preparation of paraffin sections and HE staining

After complete fixation of the tissue, the samples were placed in the embedding frame and 4 μm paraffin sections were prepared through a series of steps including dehydration, transparency, wax dipping, embedding, and continuous sectioning. The tissue sections were then subjected to histopathological analysis by staining with hematoxylin and eosin. Subsequently, the sections were observed under an Eclipse 50i microscope equipped with a camera. Images were captured using NIS-Elements 2.30 software.

### 2.6 Nucleic acid extraction and fluorescence quantitative PCR

The total RNA of brain tissues was extracted using the RNAiso Plus kit, and then reverse-transcribed into cDNA using the PrimeScript RT Kit. The mRNA levels of antiviral genes, such as ISG15, IFN-β, and OAS1, were determined using relative fluorescent quantitative PCR. Using β-actin as internal reference gene, the relative expression levels of these antiviral genes were calculated using the 2-^ΔΔCT^ method (Shao et al., 2018).

The total DNA of lung, spleen, kidney, brain, liver, and intestine were extracted using the Coffett universal genomic DNA kit. The viral loads were detected using gE specific primers listed in Table 1. The viral loads were calculated by determining the logarithm of the virus copy per 0.1 gram of tissue and expressed as log10 copies/0.1 g.

**Table 1:** The primer sequences for qPCR.

Gene		Sequence	Product size/bp	Tm/°C
β-actin	F	CATCCGTAAAGACCTCTATGCCAAC	171	60
	R	ATGGAGCCACCGATCCACA		
Ahsg1	F	CTGGTCTTGCTCCTTTGTTTT	128	60
	R	TCCACGGCCAACAAAGCTAC		
Kng1	F	TCATTACTACACTGCTCCTCT	168	58
	R	CTCGGTGCAACATATACTGGT		
Bst2	F	CAGCAGGCCCGCATCAAGGAG	163	65
	R	GCAGGAACAGTGACACTTTGA		
Usp18	F	CGGGCTGGCATGAAGAAGGAA	163	60
	R	TGTCGCCATTGATCCCAGAGA		
Oas1b	F	CCCGGCCTCGAAGCTTGATAA	164	62
	R	CTTTGCCCTTGACCCCTTTAG		
Ifi47	F	TAAGTGCTTGGATTTCTGGTC	145	60
	R	CTGTGTGCGCCTATAATAGAC		
Isg15	F	GAAGATGCTGGGGGGTAACGA	149	60
	R	TAAGACCGTCCTGGAGCACTG		
Icam1	F	CTTCTTTTGCTCTGCCGCTCT	127	60
	R	TTGCCAGGTCCAGTTCCCCAA		
Irf7	F	AGTGCTGTTTGGAGACTGGCT	130	60
	R	TTCATCCAGATCCCTACGACC		
Rsad2	F	TGTCAACTACCACTTCACTCG	188	60
	R	CCAAGTATTCACCCCTGTCCT		
Cxcl1	F	CATGGCTGGGATTCACCTCAA	177	60
	R	TACTTGGGGACACCTTTTAGC		
FGF22	F	GAGATCCGTTCTGTCCGTGT	163	60
	R	AGGCGTATGTGTTGTAGCCG		
gE	F	CTTCCACTCGCAGCTCTTCT	139	65
	R	TAGATGCAGGGCTCGTACAC		

### 2.7 Enzyme-linked immunosorbent assay (Elisa)

To evaluate the immune response induced by PRV XJ delgE/gI/TK and the levels of inflammatory factors induced after challenge in the brain, portions (100 mg) of brain tissue were weighed and placed in a 2 mL microcentrifuge tube. The tube contained a sterile steel bead and 500 μL of modified RIPA buffer with Protease inhibitor cocktail. The tissues were disrupted and then centrifuged at high speed (13,000 × g) for 10 min. The tissue supernatants were stored at −80°C until use in enzyme-linked immunosorbent assay to detect gB antibody, TNF-α, and IL-6.

To evaluate the function of metabolites in PRV-induced inflammatory response. Serum samples were used for ELISA to detect TNF-α, IL-6, IL-4, and IFN-γ. All data were analyzed and plotted using GraphPad Prism 8.0 software.

### 2.8 RNA extraction library construction and sequencing and RNA-seq data analyses

Total RNA was extracted using Trizol reagent (thermofisher, 15,596,018) following the manufacturer’s procedure. The total RNA quantity and purity were analysis of Bioanalyzer 2,100 and RNA 6000 Nano LabChip Kit (Agilent, CA, USA, 5067–1,511), high-quality RNA samples with RIN number > 7.0 were used to construct sequencing library. Therefore, all RNA samples were sent to LC-Bio Technology CO., Ltd., (Hangzhou, China) for sequencing.

Remove reads containing adapters, poly A and poly G, more than 5% of unknown nucleotides (N), low quality reads (Q value ≤20). Then sequence quality was verified using FastQC including the Q20, Q30 and GC-content of the clean data. We aligned reads of all samples to the mus musculus C57BL/6 J reference genome using HISAT2 package. The mapped reads of each sample were assembled using StringTie with default parameters. Then, all transcriptomes from all samples were merged to reconstruct a comprehensive transcriptome using gffcompare software. After the final transcriptome was generated, StringTie and ballgown were used to estimate the expression levels of all transcripts and perform expression abundance for mRNAs by calculating FPKM (fragment per kilobase of transcript per million mapped reads) value.

Genes differential expression analysis was performed by DESeq2 software between two different groups. The genes with the parameter of false discovery rate (FDR) below 0.05 and |log2(FoldChange)| > = 2 were considered differentially expressed genes. The pearson correlation coefficient between two replicas was calculated to evaluate repeatability between samples. Principal component analysis (PCA) was performed with princomp function of R in this experience. Differentially expressed genes were then subjected to enrichment analysis of GO functions and KEGG pathways using cluster Profiler software (v.3.8.1)

### 2.9 Extraction, detection and analysis of metabolites

The collected samples were thawed on ice, and metabolite were extracted with 80% methanol Buffer. Briefly, 50 mg of sample was extracted with 0.5 mL of precooled 80% methanol. The extraction mixture was then stored in 30 min at −20°C. After centrifugation at 20,000 g for 15 min, the supernatants were transferred into new tube to and vacuum dried. The samples were redissolved with 100 μL 80% methanol and stored at −80° C prior to the LC–MS analysis. In addition, pooled QC samples were also prepared by combining 10 μ L of each extraction mixture.

All samples were acquired by the LC–MS system followed machine orders. Firstly, all chromatographic separations were performed using an UltiMate 3,000 UPLC System (Thermo Fisher Scientific, Bremen, Germany). An ACQUITY UPLC T3 column (100 mm*2.1 mm, 1.8 μm, Waters, Milford, USA) was used for the reversed phase separation. The column oven was maintained at 40°C. The flow rate was 0.3ml/min and the mobile phase consisted of solvent A (water, 5mM ammonium acetate and 5mM acetic acid) and solvent B (Acetonitrile). Gradient elution conditions were set as follows: 0 ~ 0.8 min, 2% B; 0.8 ~ 2.8 min, 2 to 70% B; 2.8 ~ 5.6 min, 70 to 90% B; 5.6 ~ 6.4 min, 90 to 100% B; 6.4 ~ 8.0 min, 100% B; 8.0 ~ 8.1 min, 100 to 2% B; 8.1 ~ 10 min, 2%B. A high-resolution tandem mass spectrometer Q-Exactive (Thermo Scientific) was used to detect metabolites eluted form the column. The Q-Exactive was operated in both positive and negative ion modes.

The online KEGG, HMDB database was used to annotate the metabolites by matching the exact molecular mass data (m/z) of samples with those from database. We also used a in-house fragment spectrum library of metabolites to validate the metabolite identification. The intensity of peak data was further preprocessed by metaX. Those features that were detected in less than 50% of QC samples or 80% of biological samples were removed, the remaining peaks with missing values were imputed with the k-nearest neighbor algorithm to further improve the data quality. PCA was performed for outlier detection and batch effects evaluation using the pre-processed dataset. Quality control-based robust LOESS signal correction was fitted to the QC data with respect to the order of injection to minimize signal intensity drift over time. In addition, the relative standard deviations of the metabolic features were calculated across all QC samples, and those >30% were then removed. Student *t*-tests were conducted to detect differences in metabolite concentrations between 2 phenotypes. The *p* value was adjusted for multiple tests using an FDR (Benjamini–Hochberg). Supervised PLS-DA was conducted through meta X to discriminate the different variables between groups. The VIP value was calculated. A VIP cut-off value of 1.0 was used to select important features

### 2.10 qRT-PCR analyses of gene expression

To verify the accuracy of the sequencing results, we selected 12 differential genes for RT-qPCR analysis. These genes included 10 significantly up-regulated genes and 2 significantly down-regulated genes. We calculated the relative expression of the differentially expressed genes using the 2^−ΔΔCt^ method. The primers used for the analysis were designed based on published mRNA sequences in NCBI and synthesized by Sangon Biotech Co., Ltd. (Shanghai, China), as shown in Table 1.

### 2.11 Western blotting

For protein analysis, the brains were homogenized in RIPA buffer containing protease inhibitors. The protein concentration was quantified using a BCA Protein Assay Kit. Equal amounts of total protein were resolved by sodium dodecyl sulfate-polyacrylamide gel electrophoresis and transferred onto a polyvinylidene fluoride membrane. The membrane blots were saturated with 5% BSA in PBST for 2 h at room temperature and then incubated overnight at 4°C with primary antibodies against PI3K, p-Akt, and β-actin. After incubation, the membrane was washed three times with PBST and incubated with HRP Goat Anti-Rabbit IgG(H + L). The signals were visualized with SuperSignalTM West Pico Plus Chemiluminescent Substrate. The gray intensity of proteins was measured using Image J software.

### 2.12 Statistical analysis

We performed statistical analysis using GraphPad 7.04 software and conducted one-way analysis of variance (ANOVA). All data are expressed as mean ± standard deviation. A significance level of *p* < 0.05 was considered statistically significant.

## 3 Results

### 3.1 The LD50 of mouse intracranial infection model

The gradient dilution PRV XJ virus was injected into the brain of each experimental group using intracranial injection, while the mock group was inoculated with serum-free DMEM using the same method. The mice were continuously observed for 1 week, recording the number of deaths among the experimental mice. The median lethal dose (LD_50_) was calculated using the Reed & Muench method. In group A, the mice started dying on day 2 and all were deceased by day 3 after infection. Table 2 shows that all mice in groups A to D died, and two mice in group E also died. Additionally, all the deceased mice exhibited reduced appetite. No mice in groups F and G died. Clinical symptoms typically include itching and scratching behavior, and in severe cases, the right ear may be scratched to the point of bleeding. The virus LD_50_ was calculated as 10^–4.75^/0.02 mL PRV XJ using the Reed & Muench method.

**Table 2:** Result of median lethal dose of strain XJ by intracranial injection.

Dilution	Number	Death	Survival	Accumulated number of deaths	Accumulated number of survival	Total	Fatality rate (%)
10^−1^	6	6	0	26	0	26	100.00%
10^−2^	6	6	0	20	0	20	100.00%
10^−3^	6	6	0	14	0	14	100.00%
10^−4^	6	6	0	8	0	8	100.00%
10^−5^	6	2	4	2	4	6	33.33%
10^−6^	6	0	6	0	10	10	0.00%
10^−7^	6	0	6	0	16	16	0.00%

### 3.2 PRV del gE/gI/TK protect intracranial infected mice from death

**Figure 1:**
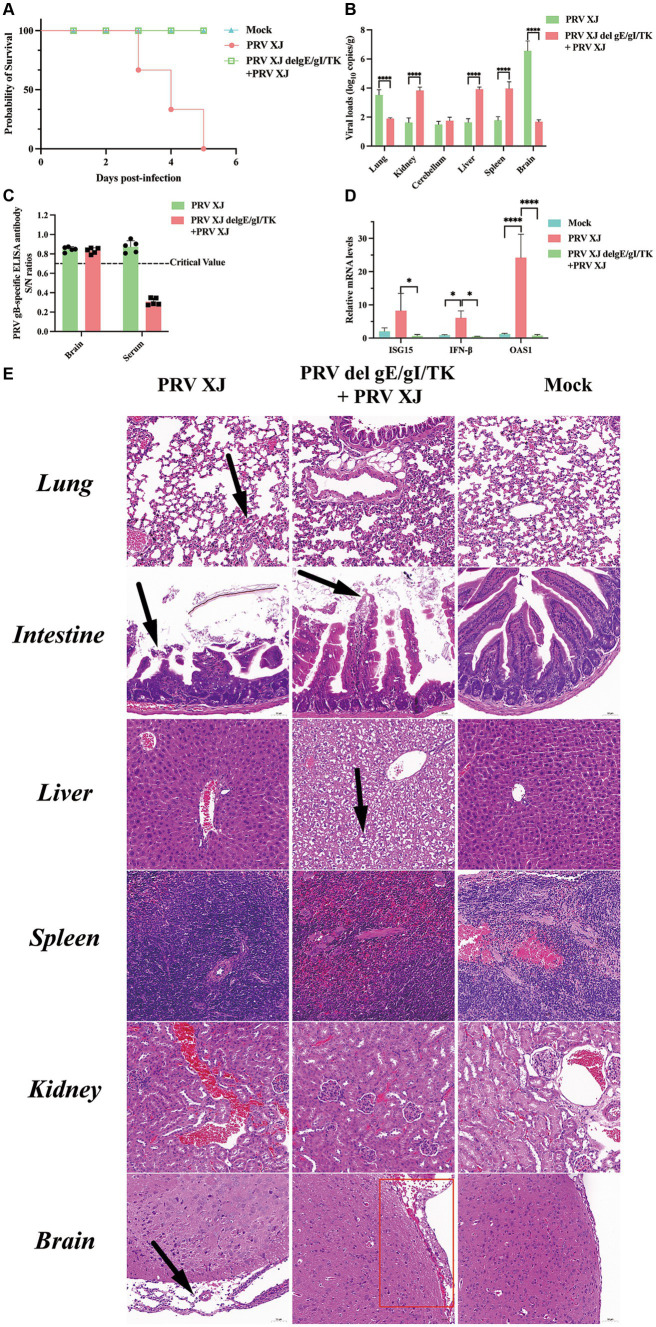
Clinical symptoms, viral loads and the histopathological lesions of mice. **(A)** Survival curves of mice in the differential treatment group. **(B)** Viral load of different tissues between the challenged group and the immunization-challenged group. **(C)** The gB-specific antibody of the brains in the PRV XJ challenged group and the immunization-challenged group. **(D)** The fold change of ISG15, IFN-β and OAS1 in the brain was determined by qRT-PCR. **(E)** Histopathological lesions of mouse among the challenged group, the immunization-challenged group and mock group (200 × magnification). Black arrows indicate lesions. Scale bars, 50 μm.

According to the LD_50_ results, the challenged group and the immunization-challenged group were injected intracranially with a dose equivalent to 10 times the LD_50_ of PRV XJ strain. The mock group was inoculated with DMEM in the same manner. Daily observations were made on the survival and clinical symptoms of the mice after the challenge. The results revealed that the challenged group exhibited the clinical symptoms included scratched the ear, decreased diet and mental depression and all mice in the challenged group died between day 3 and day 5. However, no clinical symptoms and dead were observed in the immunization-challenged group after the intracranial injection of PRV, as depicted in Figure 1A. To investigate the impact of PRV XJ delgE/gI/TK on virus proliferation in intracranial infected mice, we measured the viral loads in various organs including the brain, spleen, kidney, liver, lung, and intestine of all PRV infection mice. The results demonstrated that PRV XJ delgE/gI/TK immunization effectively reduced PRV proliferation in the brain and lungs, particularly in the brain. However, the viral loads in the kidney, liver, and spleen of the immunization-challenged mice were significantly higher than those in the challenged group (Figure 1B). These findings indicate that PRV is capable of replicating and infecting peripheral organs, such as the kidney, liver, and spleen, following intracranial infection in the immunization-challenged group. Moreover, PRV XJ delgE/gI/TK immunization demonstrated a significant inhibit ory effect on PRV proliferation in the brain of intracranial infected mice. Moreover, no gB-specific antibodies to PRV were detected in the brain of the immunization-challenged group (Figure 1C). To investigate the role of innate immunity in inducing a protective effect in the central nervous system, the mRNA levels of antiviral genes in the brain were measured. The findings revealed that during the final stage of the disease, mice in the challenged group exhibited elevated levels of antiviral genes, such as ISG15, IFN-β, and OAS1, as depicted in Figure 1D. In contrast, there was no notable difference in the mRNA levels of antiviral genes between the immunization-challenged group and the mock group.

To observe the protective effect of PRV XJ delgE/gI/TK on different tissues of mice, HE staining was used to examine the pathological changes in each group. Figure 1E shows that no noticeable lesions were observed in the mock group. In the brains of mice in the challenged group, significant meningeal edema and widening (indicated by black arrows) were observed, along with a small number of inflammatory cells in the meninges. Local congestion and widening of the meninges were also observed, and cortical neuron cells in the brains of the immunization-challenged group (highlighted in the red box) appeared wrinkled and deeply stained (indicated by black arrows). In summary, the meningeal structure in PRV infection mice was altered. In the lungs of challenged mice, local widening of the alveolar wall and slight congestion of the capillaries were observed. No significant pathological changes in the lungs were observed between the immunization-challenged group and the mock group indicating that immunization with PRV del gE/gI/TK can effectively reduce the lung damage caused by intracranial PRV infection. In the intestines, there was apical dissolution of the intestinal mucosa and exfoliation of epithelial cells in the challenged group compared to the immunization-challenged group, while the intestinal mucosa, submucosa, muscular layer, and outer membrane in the mock group remained intact without any noticeable lesions. Remarkably, the liver of the immunization-challenged group displayed obvious diffuse hepatocyte swelling, vacuolar degeneration, empty cytoplasm, nuclei mostly located in the center (indicated by black arrows), hepatic sinus stenosis, and disordered arrangement of hepatocyte cords. No noticeable lesions were observed in the liver of both the challenged group and the mock group. Additionally, no obvious lesions were found in the spleen and kidney of all mice. The boundary between the red pulp and white pulp of the spleen was clearly defined. Moreover, the structure of the renal glomerulus and renal tubules in the cortical and medullary areas appeared normal, with neatly arranged cells. These findings may be attributed to the acute death of the challenged group, which could explain the absence of more severe lesions in the liver and spleen.

### 3.3 Transcriptomic analysis

#### 3.3.1 Correlation and DEGs analysis

**Figure 2:**
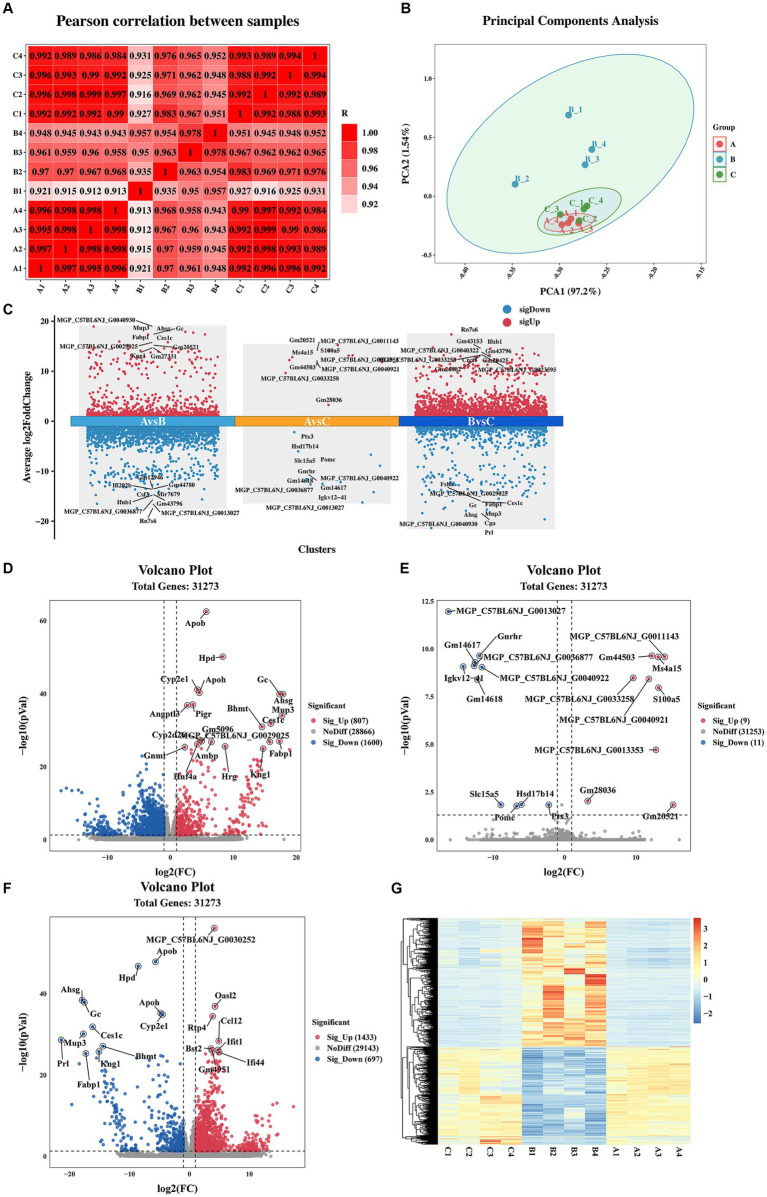
Sample correlation analysis and differential expression gene analysis. **(A)** Heat map based on Pearson correlation analysis. Data of samples with large outliers were excluded. **(B)** The principal components analysis of differential expression genes. Data of samples with large outliers were excluded. **(C)** Comparison of the differential expression genes. |Log2 (Fold change) | > 1 and *p*-value adjusted <0.05 were used to screen differentially expressed genes. **(D)** Volcano Plot of differential expression genes for the immunization-challenged group versus the challenged group. **(E)** Volcano Plot of differential expression genes for the immunization-challenged group versus the mock group. **(F)** Volcano Plot of differential expression genes for the challenged group versus the mock group. **(G)** Cluster heatmap of differential expression genes based on hierarchical cluster analysis.

Pearson correlation coefficient and PCA were utilized to assess the differences among the immunization-challenged group, the challenged group, and the mock group. As depicted in Figure 2A, the pearson correlation coefficient results demonstrated a high correlation between the duplicates in each treatment group, with correlation values exceeding 0.995, 0.935, and 0.988, respectively. Similarly, the PCA analysis revealed distinct distributions among each group (Figure 2B). Differential expression genes (DEGs) were identified and analyzed using DESeq2, based on the criteria of |log2(Fold change) | ≥ 1 and padj <0.05. Figures 2C–F illustrates that, compared to the mock group, the challenged group exhibited 1,433 up-regulated genes and 697 down-regulated genes. Moreover, the immunization-challenged group displayed 9 up-regulated genes and 11 down-regulated genes compared to the mock group. Furthermore, when compared to the challenged group, the immunization-challenged group showed 807 up-regulated genes and 1,600 down-regulated genes. These findings highlight the significant impact of PRV infection or PRV infection post-immunization on gene expression in the brain. To validate the RNA-Seq results, RT-qPCR was employed to confirm the differential expression in the brain. As presented in Supplementary Figure 1, the differential expression outcomes of the identified genes obtained through RT-qPCR and transcriptome sequencing were consistent.

Additionally, gene clustering analysis allows for the grouping of genes with similar expression patterns. The clustering results of the DEGs are depicted in Figure 2G. On one hand, the expression trends of genes within each treatment group were remarkably similar. On the other hand, the expression trends of genes in the challenged group differed significantly from those in the other two groups. Notably, the gene expression trends between the mock group and the immunization-challenged group were similar, suggesting that PRV XJ delgE/gI/TK could protect the brain from PRV.

#### 3.3.2 Go ontology and KEGG pathway analysis

The GO comprehensive database, consisting of three parts (biological process, cellular component, and molecular function), can be utilized to describe the functional properties of genes in any organism. The functional categories of DEGs based on GO analysis are displayed in Supplementary Figure 2. The DEGs between the challenged group and the immunization-challenged group primarily focus on positive regulation of RNA polymerase II transcription, innate immune response, peroxidation–reduction process, inflammatory response, and G protein-coupled receptor signaling pathway (Supplementary Figure 2A). The distribution of differential genes in the challenged group versus the mock group is similar to the immunization-challenged group versus the challenged group (Supplementary Figure 2B). The differential genes in the immunization-challenged group and the mock group are mainly enriched in obsolete oxidation–reduction process, negative regulation of NIK/NF-κB signaling, regulation of corticosterone secretion, cytochrome deposition, cell differentiation, and steroid catabolic process (Supplementary Figure 2C). This suggests that PRV XJ delgE/gI/TK may be resistant to PRV infection through delicate metabolic activities.

**Figure 3:**
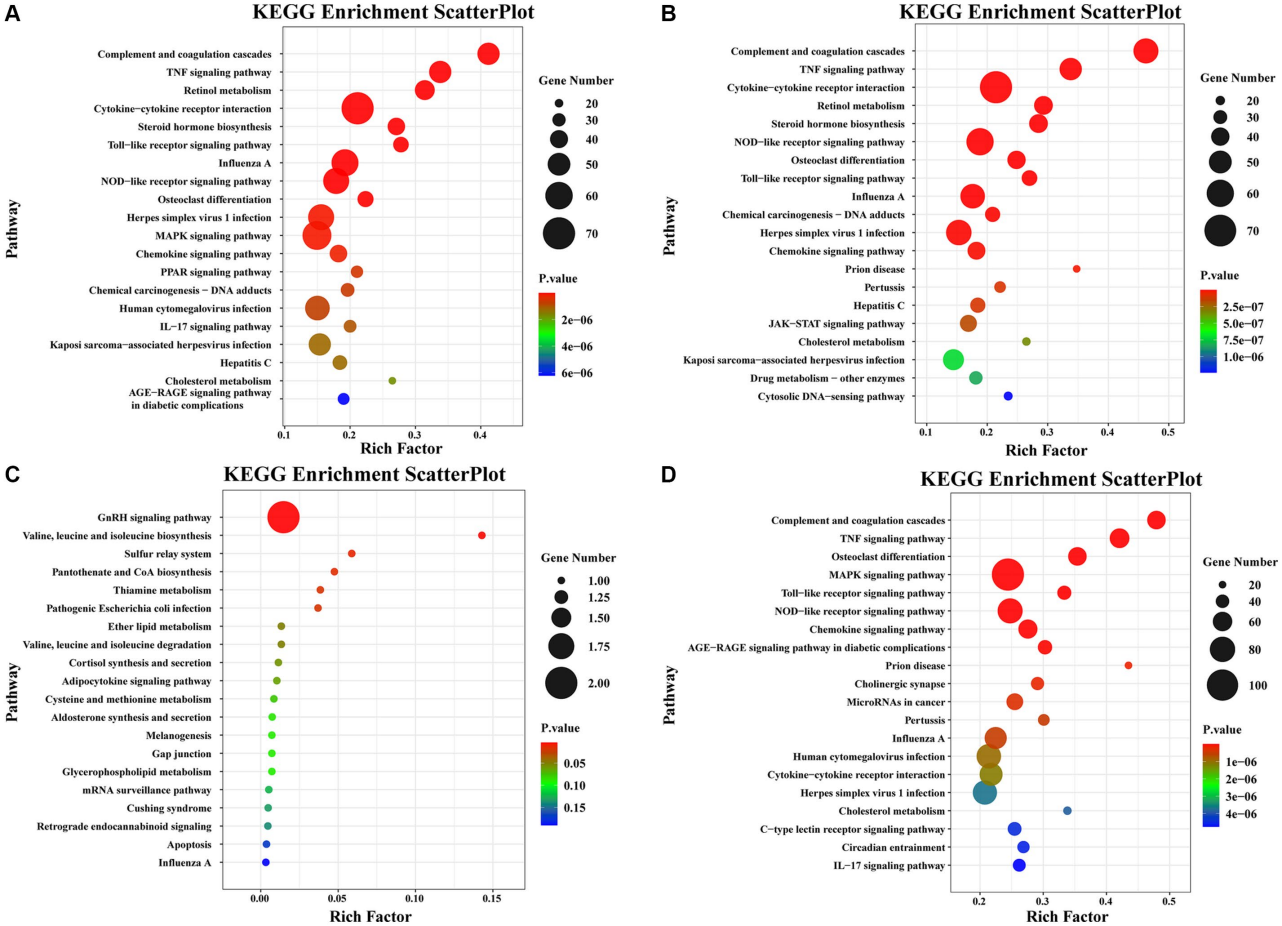
The KEGG pathway analysis of differential expression genes screened with |Log2 (Fold change) | > 1.5 and *p*-value adjusted <0.05. **(A)** Scatterplot of the KEGG pathway enriched by differential expression genes for the immunization-challenged group versus the challenged group. **(B)** Scatterplot of the KEGG pathway enriched by differential expression genes for the challenged group versus the mock group. **(C)** Scatterplot of the KEGG pathway enriched by differential expression genes for the immunization-challenged group versus the mock group. **(D)** Scatterplot of the KEGG pathway enriched by differential expression genes for the immunization-challenged group, the challenged group and the mock group.

Pathway enrichment analysis was performed on DEGs using the KEGG public database and cluster Profiler software. The top 20 pathways with the greatest enrichment are shown in Figure 3. The differential gene KEGG signaling pathway between the immunization-challenged group and the challenged group mainly enriched complement and coagulation cascades, TNF signaling pathway, retinol metabolism, cytokine−cytokine receptor interaction, steroid hormone biosynthesis, toll−like receptor signaling pathway, influenza A, NOD−like receptor signaling pathway, MAPK signaling pathway, PPAR signaling pathway, IL − 17 signaling pathway, and JAK–STAT signaling pathway (Figure 3A). The IL-17 signaling pathway, TNF signaling pathway, MAPK signaling pathway, and NOD−like receptor signaling pathway can induce inflammatory signaling pathways. While an inflammatory response is the first line of defense against the spread of viral infections, the main challenge lies in ensuring that inflammation is resolved, allowing the body’s homeostasis to return to normal. Uncontrolled inflammatory responses often lead to more severe inflammation, which can cause damage to the host. The differential gene enrichment signaling pathway further verifies that PRV XJ delgE/gI/TK may protect mice from death by regulating the level of intracranial inflammatory factors.

The KEGG signaling pathways of differentially expressed genes in the challenged group compared to the mock group were similar to those in the immunization-challenged group compared to the challenged group (Figure 3B). Furthermore, the differential genes between the immunization-challenged group and the mock group were mainly enriched in the GnRH signaling pathway, the biosynthesis of leucine (Leu), isoleucine (Ile), and valine (Val), and apoptosis signaling pathways (Figure 3C). The Leu, Ile, and Val metabolic pathways, apoptosis signaling pathways, and GnRH signaling pathway which contribute to the regulation of viral proliferation and inflammation could be involved in PRV infection. The differential genes among the immunization-challenged group, the challenged group and the mock group were mainly enriched in complement and coagulation cascades, TNF signaling pathway, osteoclast differentiation, MAPK signaling pathway, Toll-like receptor signaling pathway, NOD-like receptor signaling pathway, influenza A, human cytomegalovirus infection, cytokine-cytokine receptor interaction, herpes simplex virus 1 infection, cholesterol metabolism, and IL-17 signaling pathway (Figure 3D). Those KEGG signaling pathways of differentially expressed genes were also similar to those in the immunization-challenged group compared to the challenged group, suggesting that those differential genes are crucial for understanding the mechanisms of PRV infection and the protective role of PRV delgE/gI/TK in the central nervous system.

### 3.4 Metabolomics analysis

#### 3.4.1 Metabolite detection and differential metabolite screening and KEGG pathway analysis

**Figure 4:**
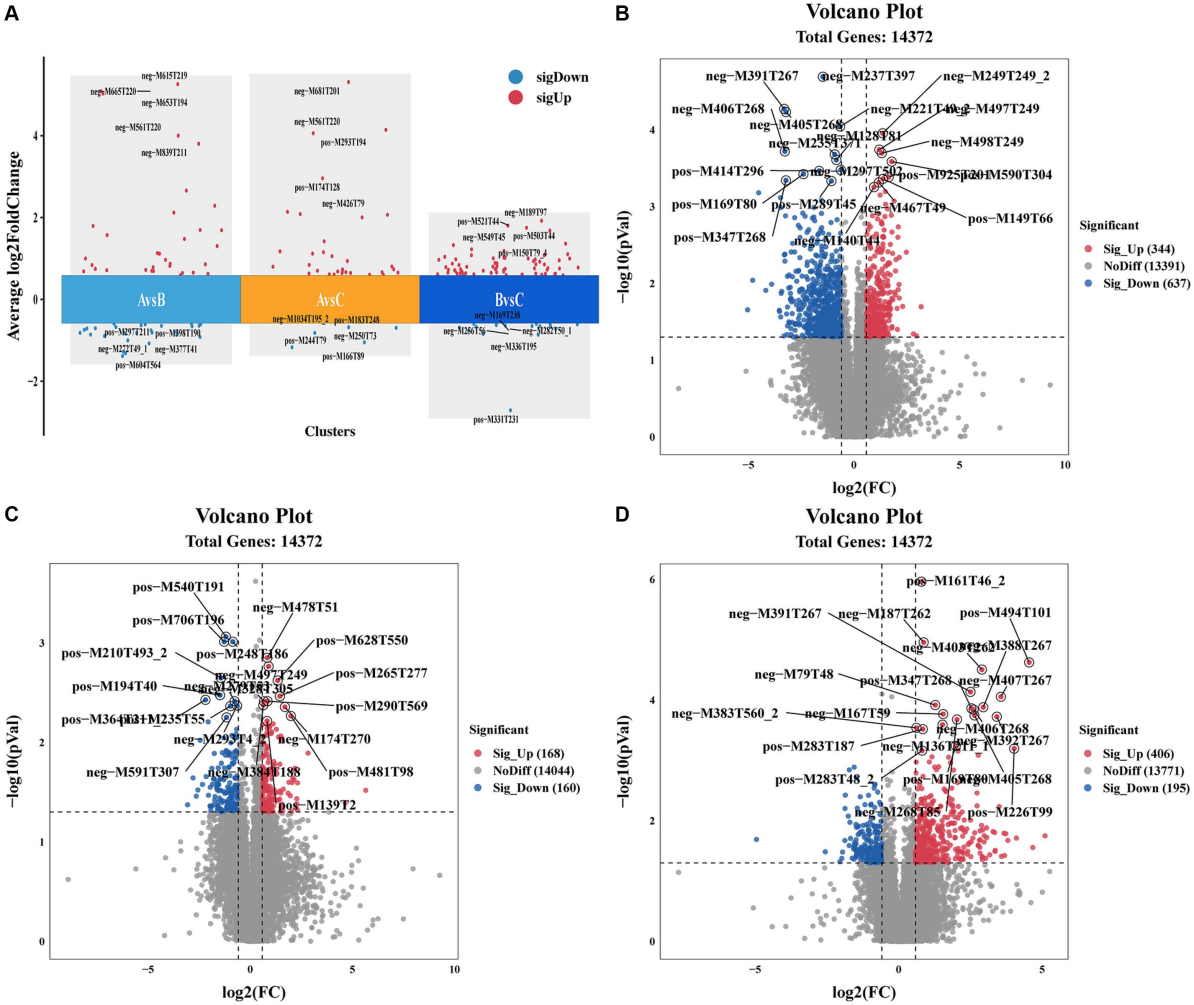
Differential metabolites analysis. **(A)** Comparison of the differential metabolites. **(B)** Volcano Plot of differential metabolites between the immunization-challenged group versus the challenged group. **(C)** Volcano Plot of differential metabolites between the immunization-challenged group versus the mock group. **(D)** Volcano Plot of differential metabolites between the challenged group versus the mock group. Each point in the volcano plot map represents a metabolite.

**Figure 5:**
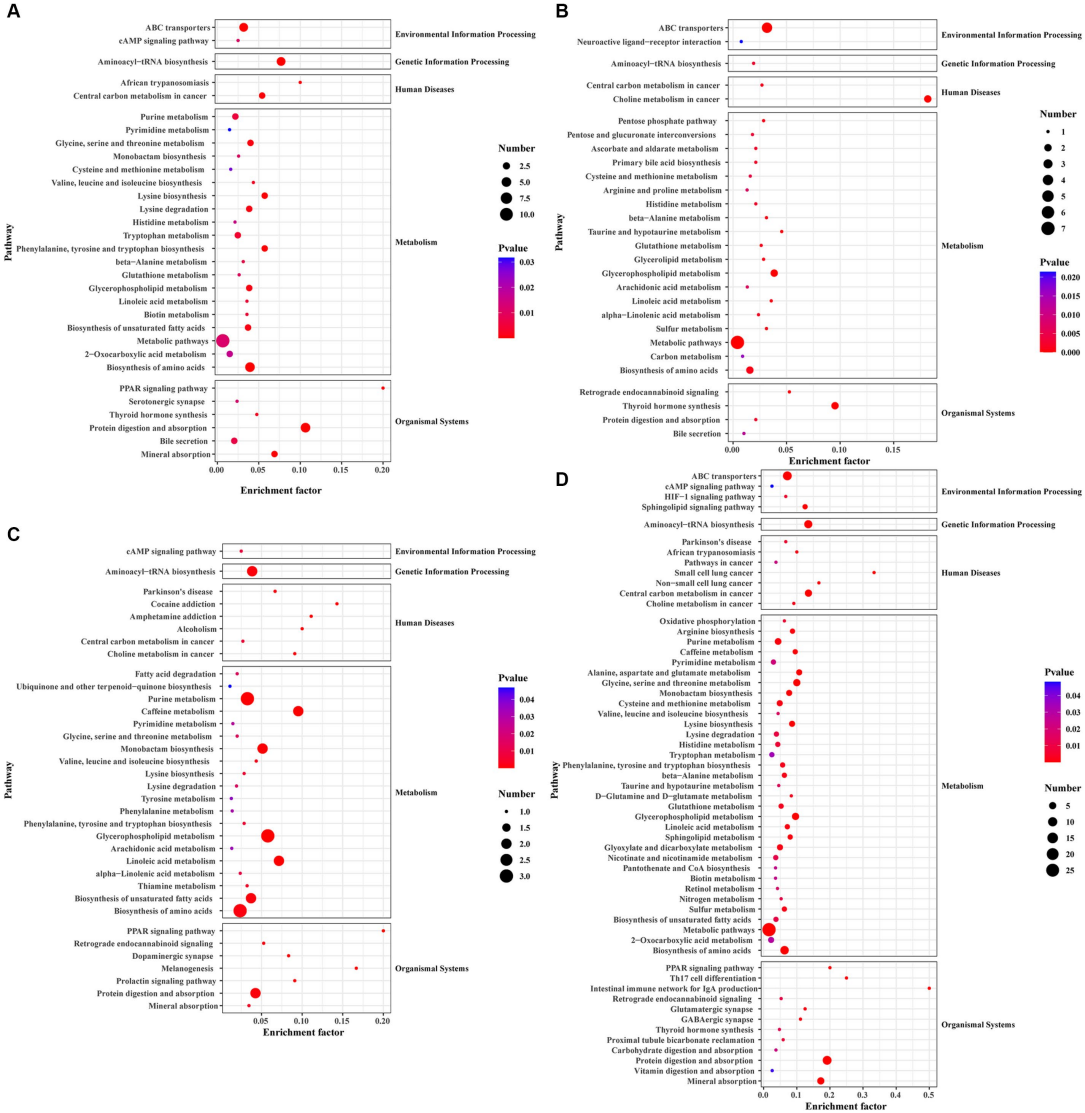
The KEGG pathway analysis of differential metabolites. **(A)** Scatterplot of the KEGG pathway enriched by differential metabolites for the immunization-challenged group versus the challenged group. **(B)** Scatterplot of the KEGG pathway enriched by differential metabolites for the challenged group versus the mock group. **(C)** Scatterplot of the KEGG pathway enriched by differential metabolites for the immunization-challenged group versus the mock group. **(D)** Scatterplot of the KEGG pathway enriched by differential metabolites for the immunization-challenged group, the challenged group and the mock group.

Molecular characteristic peaks in the samples were detected using LC–MS technology, and metabolite identification was performed using software CD3.1. The differential metabolites were screened based on VIP > 1.0, FC > 1.5, and *p*-value <0.05. The volcano plots present the screened differential metabolites. In Figure 4, there were 601 different metabolites between the mock group and the challenged group, 981 different metabolites between the challenged group and the immunization-challenged group, and 328 different metabolites between the mock group and the immunization-challenged group. Figure 5A shows the KEGG signaling pathway of the differential metabolites between the immunization-challenged group and the challenged group. The KEGG pathway mainly enriched ABC transporters, Aminoacyl−tRNA biosynthesis, Metabolic pathways, Biosynthesis of amino acids, and Protein digestion and absorption signal pathway. The differential metabolites included L-Lysine, Glutathione, L-Histidine, Indole, L-Tryptophan, Thymine, Urate, Hypoxanthine, 3-Hydroxyoctanoate, Cardiolipin, and N6-(L-1,3-Dicarboxypropyl)-L-lysine.

Differential metabolites in the KEGG signaling pathway between the challenged group and the mock group were mainly enriched in various pathways, including Aminoacyl−tRNA biosynthesis, Purine metabolism, Caffeine metabolism, Monobactam biosynthesis, Glycerophospholipid metabolism, Linoleic acid metabolism, Biosynthesis of unsaturated fatty acids, Biosynthesis of amino acids, and Protein digestion and absorption. These pathways involve metabolites such as L-Tyrosine, L-Threonine, Phosphatidylcholine, Urate, Cardiolipin, (7Z,10Z,13Z,16Z,19Z)-Docosapentaenoic acid, 7,10,13,16-Docosatetraenoic acid, 9(S)-HODE, Xanthine, and N6-(L-1,3-Dicarboxypropyl)-L-lysine (Figure 5B).

In the case of the immunization-challenged group compared to the mock group, the differential metabolites in the KEGG signaling pathway were mainly enriched in ABC transporters, Choline metabolism in cancer, Glycerophospholipid metabolism, Metabolic pathways, Biosynthesis of amino acids, Thyroid hormone synthesis, Glycerophospholipid metabolism, Arachidonic acid metabolism, Linoleic acid metabolism, and alpha−Linolenic acid metabolism. Some of the metabolites involved in these pathways include Glutathione, Taurine, L-Histidine, sn-Glycerol 3-phosphate, Phosphatidylcholine, Creatinine, and D-Xylulose 5-phosphate (Figure 5C).

The differential metabolites among the immunization-challenged group, the challenged group and the mock group were mainly enriched in PPAR signaling pathway, Retrograde endocannabinoid signaling, Glutamatergic synapse, GABAergic synapse, Thyroid hormone synthesis, Protein digestion and absorption, Vitamin digestion and absorption, Mineral absorption, Oxidative phosphorylation, Arginine biosynthesis, Purine metabolism, Pyrimidine metabolism, Alanine, aspartate and glutamate metabolism, Glycine, serine and threonine metabolism, Cysteine and methionine metabolism, Valine, leucine and isoleucine biosynthesisLysine biosynthesisLysine degradation, Histidine metabolism, Tryptophan metabolism, Phenylalanine, tyrosine and tryptophan biosynthesisbeta-Alanine metabolism, Taurine and hypotaurine metabolism, D-Glutamine and D-glutamate metabolism, Glutathione metabolism, Glycerophospholipid metabolism, Linoleic acid metabolism, Sphingolipid metabolism, Glyoxylate and dicarboxylate metabolism, Nicotinate and nicotinamide metabolism, Pantothenate and CoA biosynthesisBiotin metabolism, Retinol metabolism, Nitrogen metabolism, Sulfur metabolism, Biosynthesis of unsaturated fatty acids, Aminoacyl-tRNA biosynthesis, ABC transporters, cAMP signaling pathway, HIF-1 signaling pathway, Sphingolipid signaling pathway (Figure 5D).

### 3.5 PRV XJ delgE/gI/TK induce anti-inflammatory associated amino acid and fatty acid metabolites in the brain of mice

**Figure 6:**
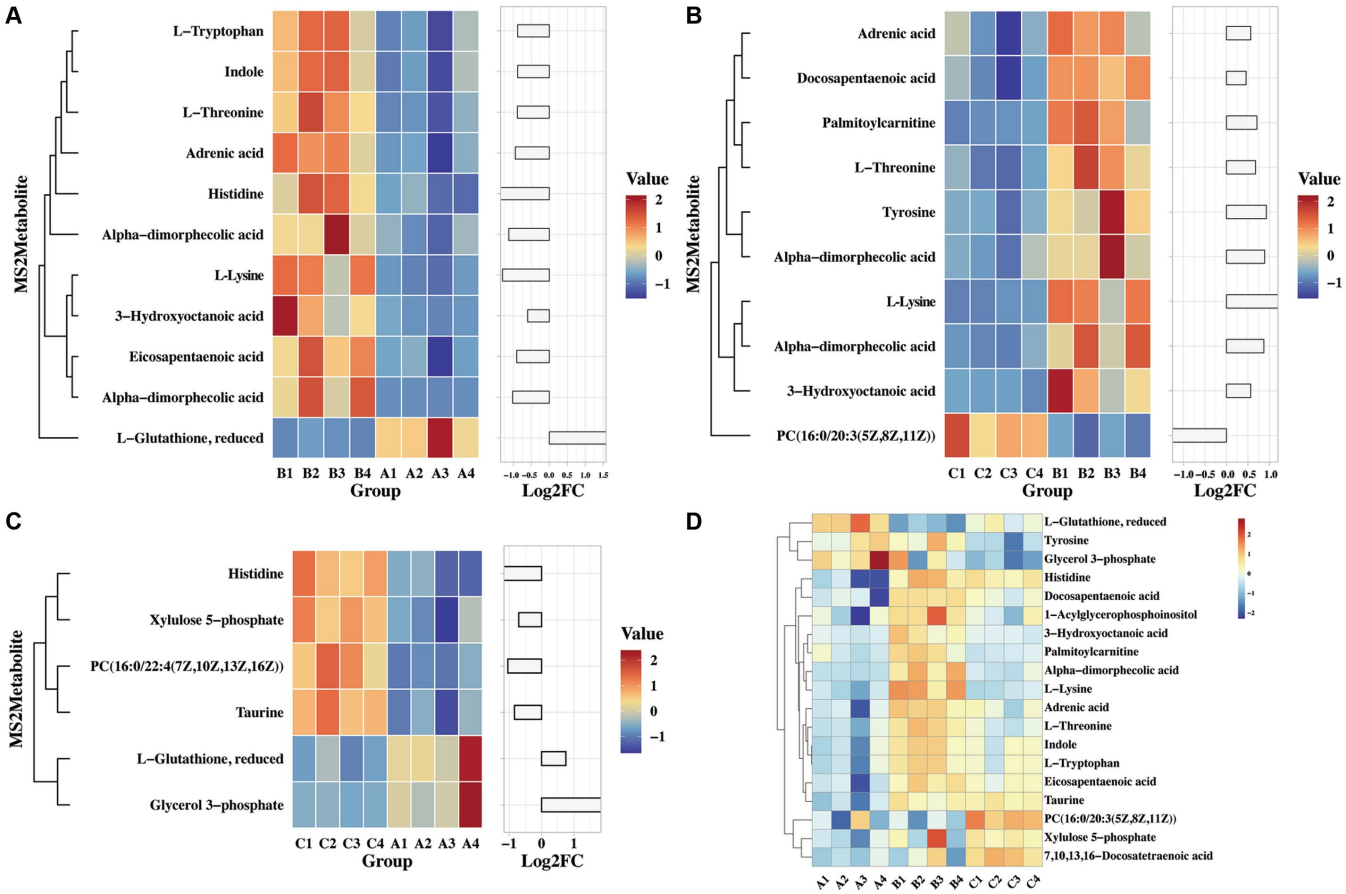
The heat map of differential MS2metabolites enriched the glycerophospholipid metabolism, linoleic acid metabolism and arachidonic acid metabolism. **(A)** The differential MS2metabolite analysis between the immunization-challenged group and the challenged group. **(B)** The differential MS2metabolite analysis between the challenged group and the mock group. **(C)** The differential MS2metabolite analysis between the immunization-challenged group and the mock group. **(D)** The relative expression levels of metabolites among the immunization-challenged group, the challenged group and the mock group.

A previous study found that viral infection triggers the production of various metabolites in the host, which can effectively combat pathogenic microorganisms. For example, Omega-3 fatty acids have the ability to deactivate enveloped viruses, Eicosapentaenoic acid can inhibit herpes virus infection, and α-linolenic acid has shown antiviral effects against influenza virus, herpes simplex virus, dengue virus, Zika virus, and SARS-CoV-2 (Thormar et al., 1987; Pfaender et al., 2013; Feng et al., 2023). In this study, we analyzed the differential metabolites that are enriched in glycerophospholipid metabolism, linoleic acid metabolism, arachidonic acid metabolism, glutathione metabolism, β-alanine metabolism, and glycine, serine, and threonine metabolism. Figure 6A illustrates significant differences in indole, threonine, 3-hydroxycaprylic acid, tryptophan, α-dimorpholinic acid, essentaric acid, epinephrine, L-lysine, 1-acylglycerol phosphate inositol, histidine, and glutathione between the challenged group and the immunization-challenged group, suggesting that the PRV XJ delgE/gI/TK may induce metabolites with anti-inflammatory properties to prevent excessive inflammation and subsequent death in mice.

As shown in Figure 6B demonstrates that PRV induced high levels of various metabolites, including threonine, 3-hydroxycaprylic acid, α-dimorolinic acid, docosapentaenoic acid, epinephrine, L-lysine, 1-acylglycerophosphate inositol, tyrosine, and phosphatidylcholine, compared to the mock group. Additionally, PRV significantly reduced palmitoyl carnitine levels, which can inhibit the activity of glutamate transporter 2. It is possible that after infecting the central nervous system, PRV regulates glutathione synthesis by modulating the activity of glutamate transporter 2, thereby suppressing the production of anti-inflammatory factors.

Furthermore, compared to the mock group, the immunization-challenged group exhibited a significant reduction in metabolites such as xylulose 5-phosphate, taurine, histidine, and glycerol 3-phosphate, while L-glutathione and phosphatidylcholine with anti-inflammatory properties were up-regulated (Figure 6C). The relative expression levels of those metabolites in the three groups were compared. As shown in Figure 6D, it was found that there were differences in reduced glutathione among the three groups. In conclusion, these amino acid and fatty acid metabolites are strongly associated with PRV XJ delgE/gI/TK-induced immune protection in the central nervous system.

### 3.6 PRV XJ delgE/gI/TK regulates inflammation by inducing fatty acid metabolites

**Figure 7:**
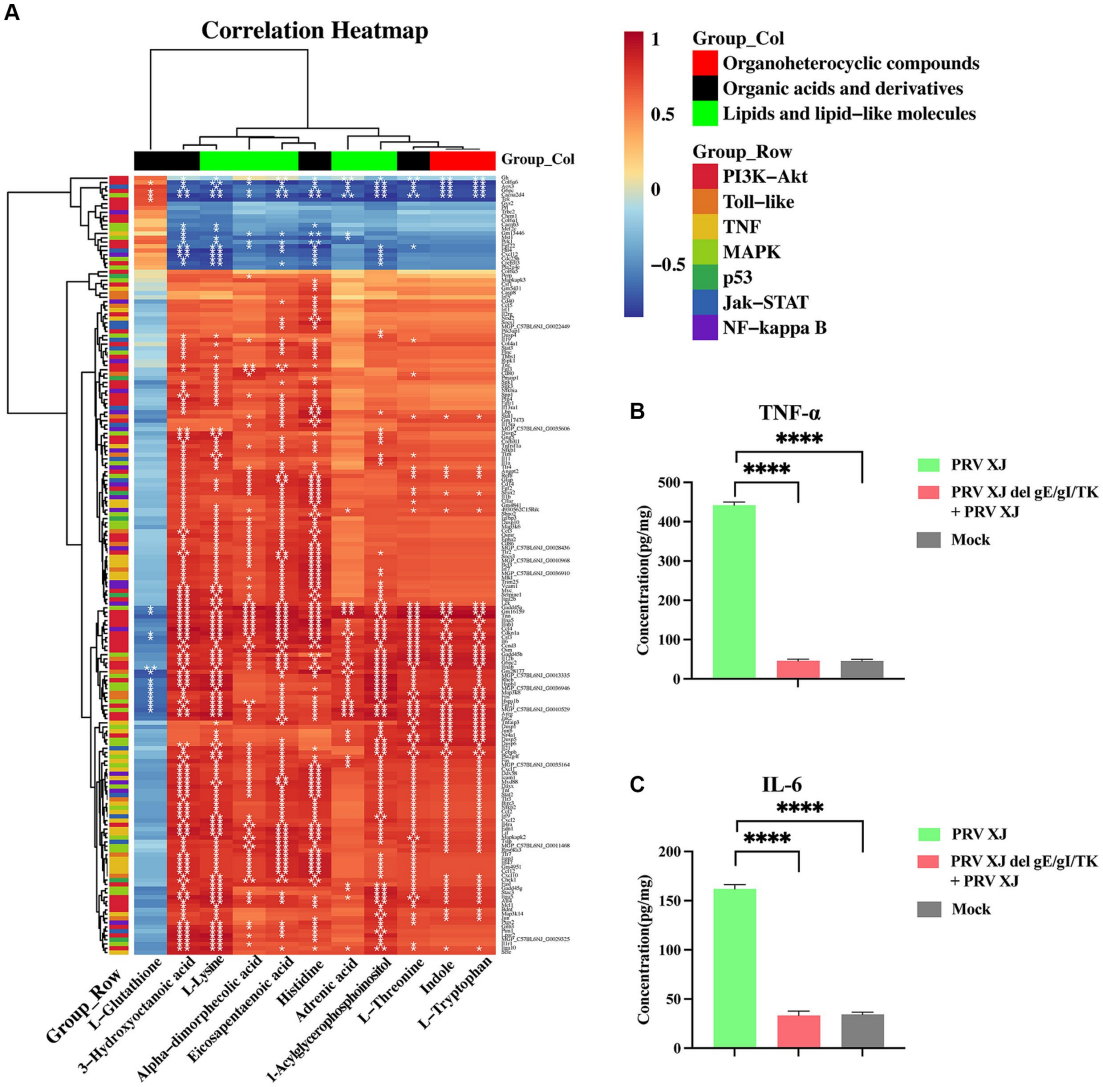
The correlation analysis and the inflammatory factor level. **(A)** The correlation analysis of the organoheterocyclic compounds, organic acids and derivatives and lipids and lipid-like molecules between the immunization-challenged and the challenged group. **(B, C)** The IL-6 and TNF-α levels of the brains. The significance of differences was analyzed by One-way ANOVA. ****p < 0.0001. The data in this figure represent the mean ± SD.

The transcriptome and metabolome of the brain were jointly analyzed. In metabolomics, we focused on glycerophospholipid metabolism, linoleic acid metabolism, and arachidonic acid metabolism. In the transcriptome, our focus was on the PI3K-Akt, Toll-like receptor signaling pathway, MAPK, p53, Jak–STAT, NF-κB, and TNF signaling pathway. We performed correlation cluster analysis to examine the relationship between important metabolites and inflammatory response and innate immune signaling pathways. Figure 7 shows the results, indicating that glutathione is correlated with the PI3K-Akt signaling pathway, MAPK signaling pathway, and toll-like receptor signaling pathway. Other organic acids and their derivative metabolites are significantly correlated with all of the above transcriptome signaling pathways. In terms of organic heterocyclic compounds, indole and L-tryptophan are mainly correlated with the PI3K-Akt, Toll-like receptor signaling pathway, TNF, MAPK, and JAK–STAT signaling pathways. Lipids and lipid molecules, such as α-linoleic acid, esentaric acid, L-lysine, and 1-acylglycerophosphate inositol, are significantly correlated with all of the above transcriptome signaling pathways. Additionally, adrenergic acid is significantly correlated with the PI3K-Akt and MAPK signaling pathways. In conclusion, these results suggest that those metabolites are involved in the signaling pathways related to inflammation and innate immune response.

To further understand the changes in inflammatory response, the levels of inflammatory factors in the brains were measured using the ELISA method (Figures 7B, C). It was found that TNF-α and IL-6 were significantly upregulated in the challenged group. However, there was no significant difference in the levels of these inflammatory factors between the brains of PRV XJ delgE/gI/TK mice and the control group after immunization. This finding suggests that PRV XJ delgE/gI/TK may protect mice from death caused by intracranial infection of PRV XJ strains by upregulating anti-inflammatory metabolites.

### 3.7 Inhibiting the inflammatory response effectively protected mice from PRV-induced death

**Figure 8:**
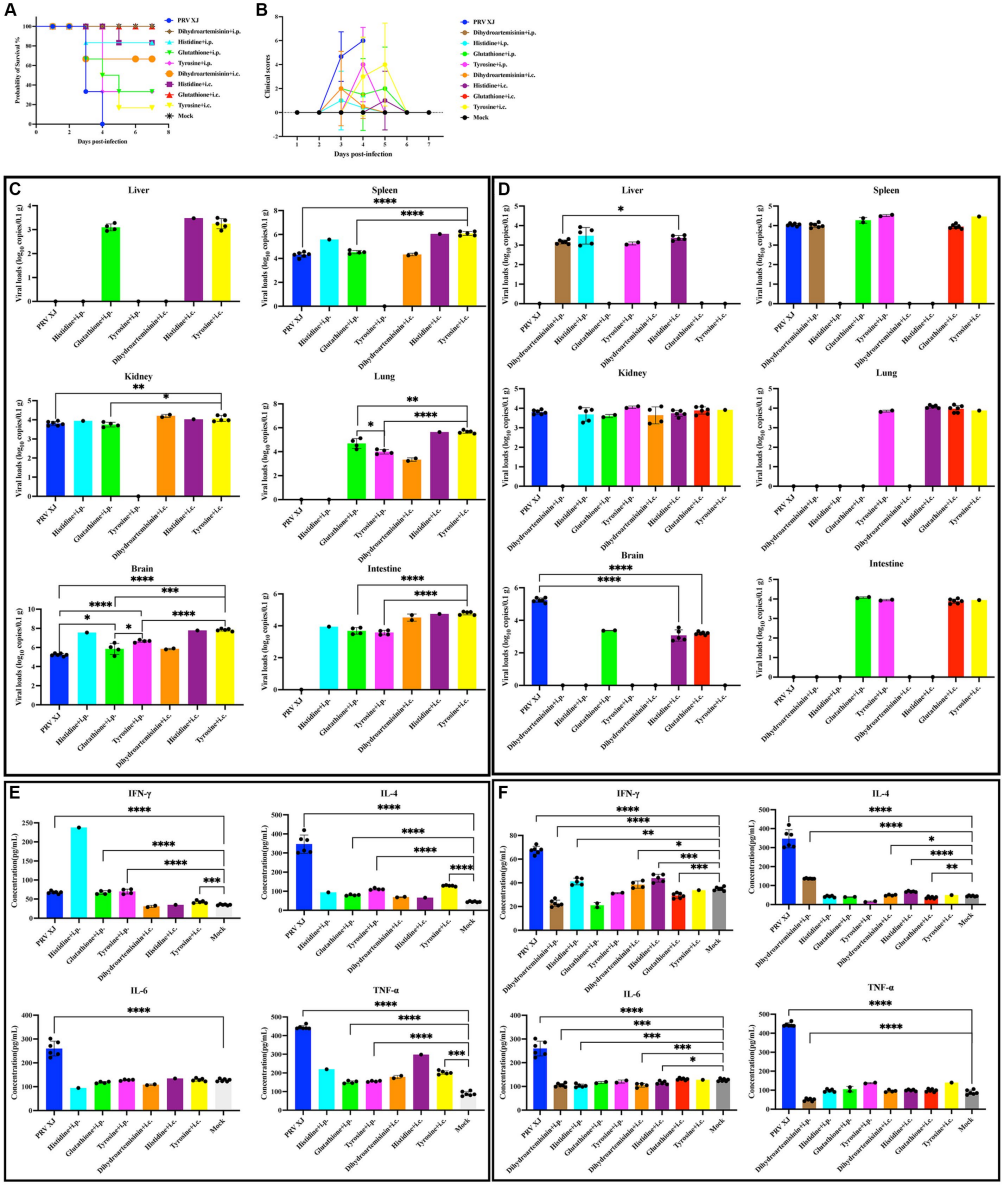
Clinical symptoms, viral loads and serum cytokine levels among the metabolites treated group, the challenged group and the mock group. **(A)** Survival curves of mice in the differential treatment group. **(B)** The clinical scores during the experiment period. **(C)** The viral loads of the PRV-challenged group and dead mice in the metabolites treated group. **(D)** The viral loads of the PRV-challenged group and non-dead mice in the metabolites treated group. No significance analysis was performed for the group with the number of mice less than 3. **(E)** The serum IL-4, INF-γ, IL-6 and TNF-α levels of the PRV-challenged group and dead mice in the metabolites treated group. **(F)** The serum IL-4, INF-γ, IL-6 and TNF-α levels of the PRV-challenged group and non-dead mice in the metabolites treated group.

Based on the correlation analysis of important differential metabolites and differentially expressed genes, it is speculated that PRV XJ delgE/gI/TK strains may protect mice from death caused by PRV XJ by inhibiting the inflammatory response through the regulation of metabolites. We screened four metabolites that regulate inflammation including tyrosine, histidine, glutathione, and dihydroartemisinin to investigate their protective effects on PRV-intracranial infection in mice. As shown in Figures 8A, B, mice in the challenged group displayed obvious clinical symptoms and died starting from day 3, with all mice dead by day 4. However, no deaths or clinical symptoms were observed in the glutathione i.c group and the dihydroartemisinin i.p group. On the other hand, death and obvious clinical symptoms were observed in the other metabolite treatment groups. In the histidine i.p group and the histidine i.c group, one mouse each died. The tyrosine i.p group had the highest number of deaths compared to the other metabolite treatment groups. These results indicate that intracranial injection of glutathione and intraperitoneal injection of dihydroartemisinin could completely protect mice from death caused by PRV infection in the central nervous system. Additionally, intracranial or intraperitoneal injection of histidine also effectively protected mice from death caused by PRV-intracranial infection.

To investigate the protective effect of metabolites on PRV-intracranial infected mice, we examined the virus loads in various organs of mice from the challenged group and the metabolite treatment group. Within the same treatment group, there was a significant difference in viral load between the deceased mice and the surviving mice. Therefore, the mice in the PRV challenge group were compared with the deceased mice or surviving mice in the metabolite treatment group, respectively. As depicted in Figure 8C, the results revealed that the viral loads in various tissues of dead mice in the glutathione i.p group and tyrosine i.c group were significantly higher than those in the challenged group. Additionally, the viral loads in the brains of dead mice in the glutathione i.p group, tyrosine i.p group, and tyrosine i.c group were higher than that in the challenged group. In Figure 8D, the viral loads in the brains of all surviving mice were significantly lower than that of the challenge group. PRV was also detected in other tissues of surviving mice. And there were high levels of viral loads in other tissues of mice in the glutathione i.c group. Moreover, intracranial injection of dihydroartemisinin, intracranial injection of histidine, and intraperitoneal injection of histidine can significantly reduce the proliferation of the virus in the spleen, while intraperitoneal injection of dihydroartemisinin can significantly reduce the colonization of the virus in the kidneys. These results suggest that intraperitoneal injection of dihydroartemisinin or histidine, as well as intracranial injection of dihydroartemisinin, histidine, or glutathione, can effectively reduce the viral loads of PRV in the central nervous system, although complete virus removal is not achieved. Furthermore, the different injection methods of metabolites also had an impact on mouse survival and virus colonization.

In order to investigate the impact of metabolites treatment on the PRV-induced inflammatory response and immune response, the serum levels of TNF-α, IL-6, IL-4, and IFN-γ were measured. Notably, there were notable differences in cytokine levels between dead and non-dead mice within the same treatment group. Therefore, the mice in the PRV challenge group were compared with the deceased mice or surviving mice in the metabolite treatment group, respectively. Figure 8E demonstrates that the levels of IFN-γ, IL-4, and TNF-α in the glutathione i.p group, the tyrosine i.p group, and the tyrosine i.c group were significantly higher than those in the mock group, indicating that the dead mice in these metabolite treatment groups exhibited elevated levels of inflammatory response and innate immunity. Figure 8F reveals that compared to the mock group, the levels of IFN-γ in the dihydroartemisinin i.p group and glutathione i.c group were significantly lower, while the levels of IFN-γ in the histidine i.p group, dihydroartemisinin i.c group, and histidine i.c group were significantly higher but still lower than the mock group. Moreover, the levels of IL-4 in the dihydroartemisinin i.c group, dihydroartemisinin i.p group, and histidine i.c group were significantly higher than those in the mock group, whereas the level of IL-4 in the glutathione i.c group was significantly lower. These findings suggest that the excessive immune response gradually returned to normal levels in the surviving mice. Additionally, the IL-6 levels in the dihydroartemisinin i.p group, histidine i.p group, dihydroartemisinin i.c group, and histidine i.c group were significantly lower compared to the mock group. There was no significant difference in the IL-6 levels between the glutathione i.c group and the mock group. The TNF-α levels in the dihydroartemisinin i.p group were significantly lower than those in the mock group. There was no significant difference in the TNF-α levels among the histidine i.p group, dihydroartemisinin i.c group, histidine i.c group, glutathione i.c groups, and the mock group. The results showed that the levels of inflammatory factors returned to normal in the surviving mice. In conclusion, our results suggest that intraperitoneal injection of dihydroartemisinin and intracranial injection of glutathione can effectively inhibit the inflammatory response and protect against death caused by PRV infection in the central nervous system.

**Figure 9:**
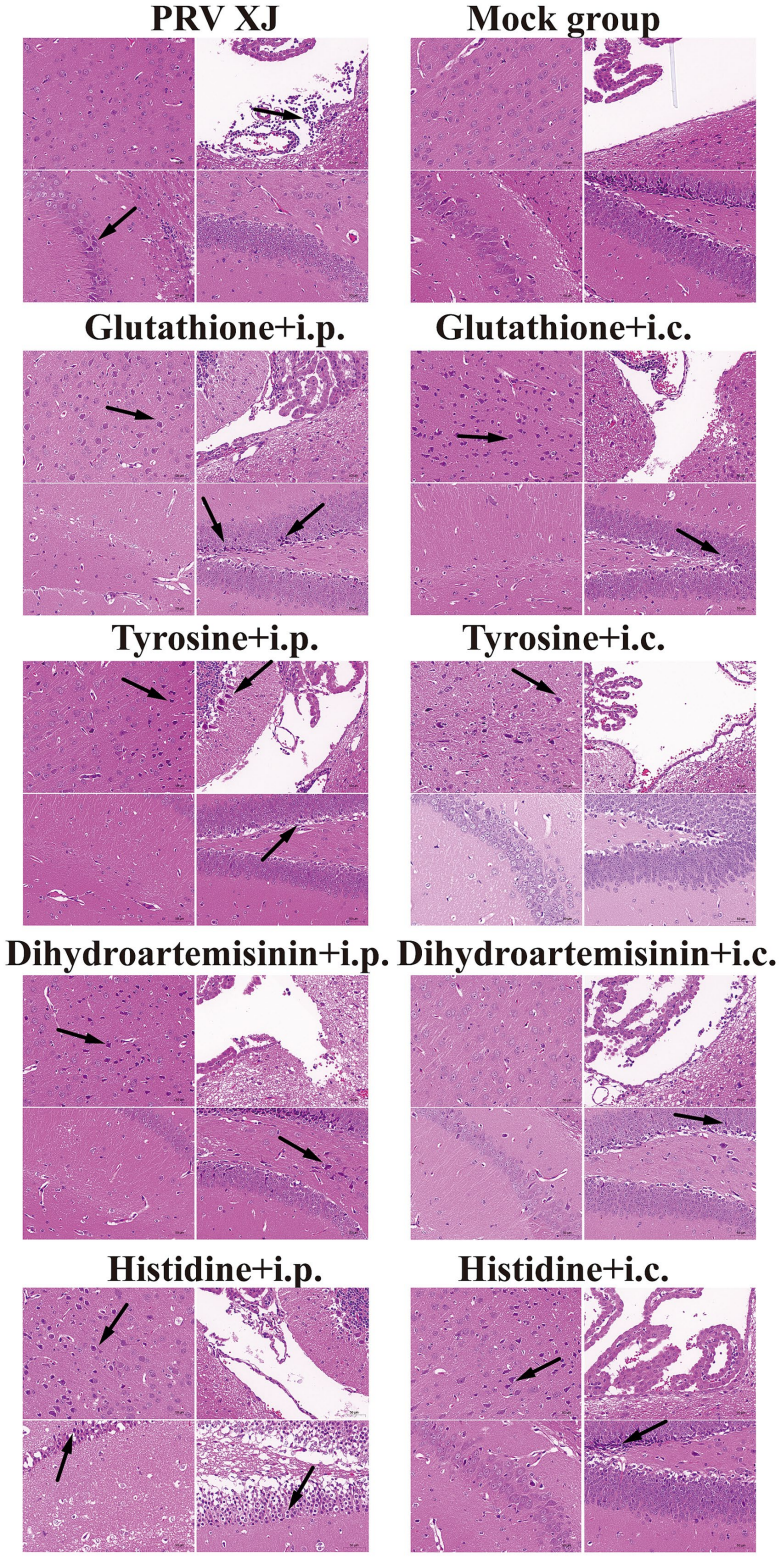
Histopathological lesions of mice among the challenged group, the metabolites treatment group and mock group (200 × magnification). Black arrows indicate lesions. Scale bars, 50 μm.

In the challenge group, there was clear infiltration of inflammatory cells in the fourth ventricle, while no inflammatory reaction was observed in any of the metabolite treatment groups. The changes in the prefrontal cortex were more pronounced in the glutathione i.c group, the tyrosine i.c group, the dihydroartemisinin i.p group, the histidine i.c group and the histidine i.p group. Similarly, the changes in the hippocampus were more evident in the glutathione i.p group, the dihydroartemisinin i.p group, the dihydroartemisinin i.c group, the tyrosine i.p group, the histidine i.c group and the histidine i.p group. These findings indicate that metabolite treatment protected the mice by suppressing the inflammatory response caused by PRV infection. However, it should be noted that the damage to the central nervous system caused by pseudorabies cannot be fully reversed in the short term after metabolite treatment (Figure 9).

### 3.8 PRV XJ delgE/gI/TK regulates inflammation through the PI3K/Akt signaling pathway

**Figure 10:**
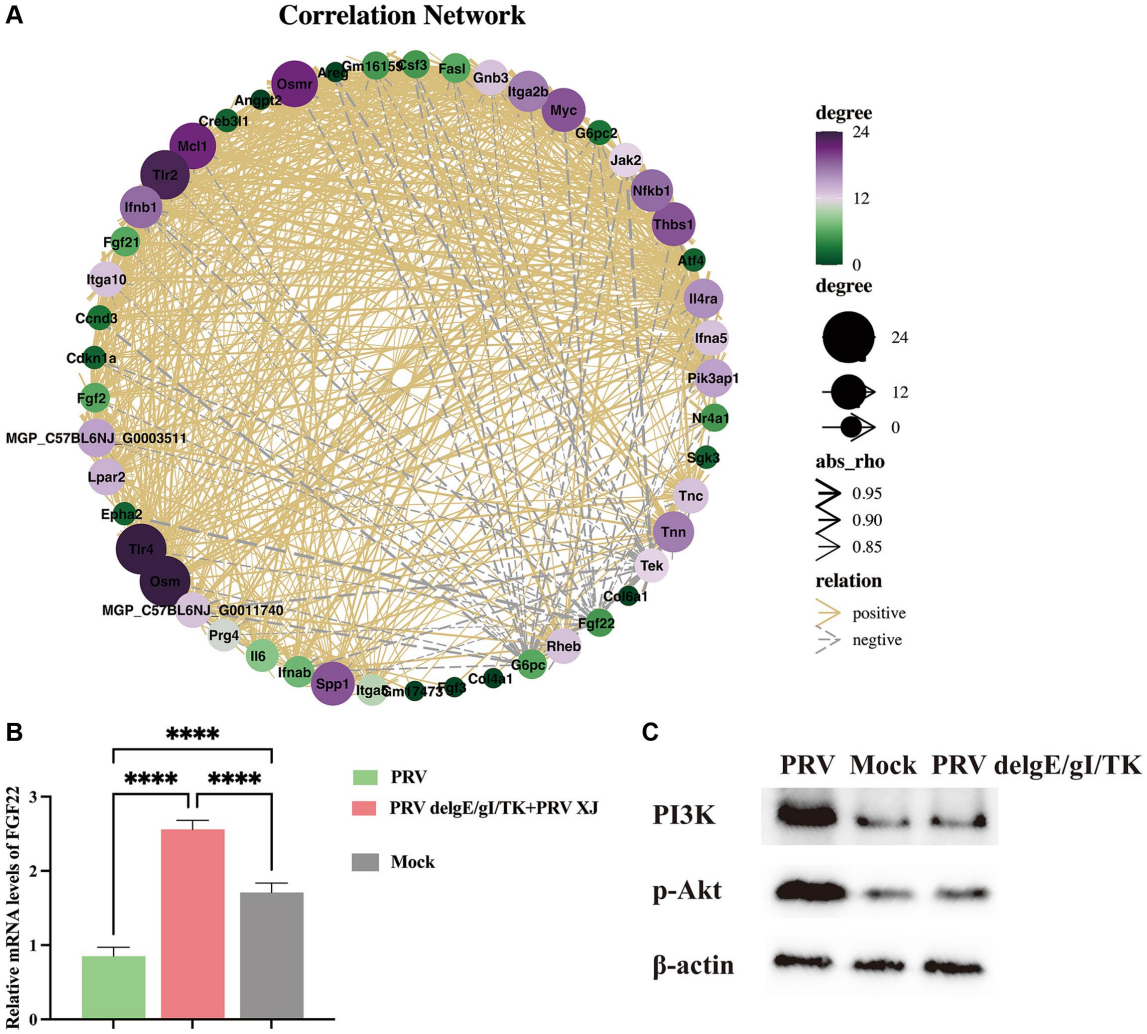
The correlation Network analysis and verification. **(A)** The correlation Network of the inflammatory signaling pathways, the glycerophospholipid metabolism, linoleic acid metabolism and arachidonic acid metabolism between the challenged group and the immunization-challenged group. **(B)** The FGF22 relative mRNA expression among the challenged group, immunization-challenged group and the mock group. **(C)** The protein levels of the PI3K/Akt detected by western blotting.

Metabolites such as 5Z, 8Z, 11Z, 14Z, 17Z-eicosapentaenoic acid, 7,10,13, 16-docosatetraenoic acid, 3-hydroxycaprylate, glutathione, L-histidine and L-lysine are involved in the regulation of inflammatory response (Chen et al., 2024), suggesting that PRV XJ delgE/gI/TK immunization may regulate inflammatory response through metabolites. Subsequently, we performed a co-expression network analysis of important metabolites and genes associated with inflammatory response between the immunization-challenged group and the challenged group. Genes and metabolites with |Rho| > 0.8 and *p* value<=0.01 to establish the related network, which was visualized using the Cytoscape software. The results indicate that histidine, lysine, and 3-Hydroxyoctanoic acid play crucial roles as central nodes in interacting with numerous genes in inflammatory signaling pathways (Figure 10A), suggesting that metabolites may play a significant role in the regulation of inflammatory responses.

According to our network analysis, we found that FGF22 expression was higher in the immunization-challenged group compared to the challenged group. FGF22 belongs to the Fibroblast Growth Factor family and is closely related to FGF3, FGF7, and FGF10. Fibroblast Growth Factor Receptors (FGFRs) are known to activate various signaling pathways, including PI3K/Akt, phospholipase C/protein kinase C, and Ras/Raf/ERK pathways (Laskaratos et al., 2021). FGF22 specifically activates FGFR2b and FGFR1b, leading to the activation of downstream signaling pathways such as MAPK/ERK and AKT by FRS2, AKT by PI3K, or IP3/DAG/Ca2+ by PLCγ (Dabrowski et al., 2015). The PI3K/AKT signaling pathway is known to regulate inflammatory response. However, the role of the FGF22/PI3K/AKT signaling pathway in the inflammatory response induced by PRV is still unknown. To investigate this further, we examined the expression of FGF22 gene and PI3K/Akt signaling pathway-related genes in each group. As depicted in Figure 10B, our results showed that FGF22 expression was significantly lower in the challenged group compared to the mock group, but significantly higher in the immunization-challenged group. This suggests that the FGF22 gene plays a crucial role in PRV XJ delgE/gI/TK resistance to viral infection. Additionally, we observed that p-PI3K and p-Akt were significantly up-regulated in the challenged group compared to the mock group, indicating activation of the PI3K/Akt signaling pathway following PRV infection. However, there was no significant difference in the immunization-challenged group (Figure 10C). These findings suggest that the PI3K/Akt signaling pathway may contribute to host defense against PRV.

## 4 Discussion

Since late 2011, variant PRV strains have emerged from Bartha-K61-vaccinated pig farms in China, causing significant economic losses to the pig industry. However, the classical attenuated vaccine (strain Bartha-K61) has not been effective in providing sufficient protection against the prevalent PRV variants (Gu et al., 2018). Recombination events have been identified in the field, including recombination between Bartha-K61 and PRV variant, recombination among PRV variant strains, and recombination between PRV type I and PRV type II strains (Huang et al., 2020; Lian et al., 2023; Qin et al., 2023). Notably, reports of natural TK gene deletion have confirmed that live virus vaccines can contribute to an increase in strain diversity, making control more challenging. Currently, there are various gene deletion strains based on mutant strains that can effectively protect newborn piglets from death caused by variants or classic strains (Zhang et al., 2023). Treatment options for PR are currently lacking, and the primary methods for prevention and control of PRV are vaccination, which mainly includes attenuated vaccine and inactivated vaccine. Vaccination is the most effective means of preventing the large-scale spread of PR. However, live vaccines may lead to increased strain diversity and accelerate the risk of strain mutation. On the other hand, inactivated vaccines also have some limitations, such as a weak ability to induce natural immunity, a long time to release the virus, and strong immune side reactions. Therefore, this study utilized a mouse intracranial infection model of PRV to screen for differential metabolites and differential expression genes, aiming to explore the mechanism by which PRV XJ delgE/gI/TK regulate intracranial genes or metabolites to protect the central nervous system. The results of this study provide a theoretical basis for the development of new anti-PRV drugs.

Li Xue et al. investigated the impact of PRV infection on various cellular pathways. They observed that PRV infection led to the activation of p65 phosphorylation, stimulation of NF-κB, induction of inflammatory factors, and up-regulation of cytokines such as IFN-β, TNF-α, IL-1β, IL-6, and IL-18, resulting in a cytokine storm (Li et al., 2022; Chen et al., 2023). The study also highlighted the role of MCP-1, which is primarily induced by endogenous inflammatory factors like IL-1 and TNF-α. This induction can trigger the production of multiple cytokines, leading to a vicious cycle (Ogando et al., 2003). Furthermore, PRV US3 protein was shown to promote axon translation through the PI3K/Akt-mToRC1 signaling pathway, facilitating effective retrograde transport of nucleocapsid to the cell body early after virus entry (Esteves et al., 2022). Additionally, ERK1/2, a component of the MAPK signaling pathway, was found to mediate various cellular processes and could be induced by PRV UL46, potentially impacting cell survival and apoptosis (Schulz et al., 2014). Moreover, PRV infection significantly activated the NLRP3 inflammasome, while also up-regulating JAK–STAT and NOD-like signaling pathways that regulate the levels of IL-6 and TNF-α (Li et al., 2022). Transcriptome analysis revealed the activation of TNF signaling, NOD-like receptors, JAK–STAT, MAPK, and IL-17 signaling pathways post-PRV infection, all of which play crucial roles in mediating inflammation. Shangguan et al. (2022) corroborated these findings, demonstrating the activation of these signaling pathways involved in apoptosis, inflammatory response regulation, and viral proliferation.

3-hydroxycaprylate, a metabolite of medium chain fatty acid oxidation, plays a significant role in regulating the inflammatory response. Studies have shown that using 3-hydroxycaprylate as a dressing can effectively inhibit inflammation in wound areas, demonstrating its strong anti-inflammatory effect (Seta et al., 2022). Furthermore, arginine, an essential amino acid for certain herpes viruses, can inhibit virus proliferation through accelerated catabolism (Pedrazini et al., 2022). Following tissue injury, the body produces various inflammatory mediators, including prostaglandins and leukotrienes. These mediators cause blood vessel dilation, tissue swelling, and infiltration of white blood cells. Leukotrienes, catalyzed by 5-lipoxysynthase, are crucial mediators in the inflammatory response, promoting inflammatory cell infiltration and activation. Hence, 5-lipoxygenase plays a vital role in regulating inflammation. (5Z, 8Z, 11Z, 14Z, 17Z)-eicosapentaenoic acid acts as a 5-lipoxygenase inhibitor to regulate inflammation. 7,10,13,16-docosatetraenoic acid, also known as adrenic acid, is involved in the metabolism of α-linolenic acid and linoleic acid. It is a natural polyunsaturated fatty acid that can be metabolized by cells into amphiprostaglandins and diisoepoxyacetic acid (diisoEETS), which function as prostaglandin inhibitors (Ren et al., 2006). Histidine possesses anti-inflammatory and antioxidant effects, primarily achieved through the activation of NF-κB and PPARγ (Feng et al., 2013; Sun et al., 2014). Adiponectin, a protein secreted by adipose tissue, plays a role in reducing inflammation. Histidine exhibits anti-inflammatory properties by inhibiting NF-κB activation, suppressing the production of pro-inflammatory factors, and stimulating the secretion of adiponectin via PPARγ activation. Previous studies have demonstrated the beneficial antiviral and anti-inflammatory functions of amino acid metabolites and lipid metabolites. In this study, notable differences in metabolites were observed between the challenged group and the immunization-challenged group. Furthermore, correlation analysis revealed a strong association between the histidine, L-lysine, and 3-hydroxycaprylate metabolites and the KEGG pathway related to the inflammatory response. These findings further support the notion that up-regulated glutathione by PRV XJ delgE/gI/TK can effectively safeguard mice against PRV-induced mortality.

Fgf22 and its receptors Fgfr1b and Fgfr2b are consistently expressed in the central nervous system and are important endogenous regulators of synaptic formation. Compared with the challenged group, FGF22 was significantly upregulated in the immunization-challenged group, suggesting that FGF22 may play an important role in anti-PRV. Activation of the PI3K/AKT signaling pathway is common in a variety of human diseases, including cancer, diabetes, cardiovascular disease, and neurological diseases, and plays an important role in the regulation of apoptosis and autophagy. In this study, it was found that the PI3K/AKT signaling pathway was significantly up-regulated after PRV infection, and the immunization-challenged group mice was consistent with that in the mock group, indicating that the PI3K/AKT signaling pathway plays an important role in PRV infection, but the mechanism needs to further study.

In this study, we established a mouse intracranial infection model of PRV and analyzed the transcriptome and metabolome of the mouse brain. Transcriptomics revealed differential expression of various inflammation-related signaling pathways and innate immune signaling pathways, including the TNF signaling pathway, toll-like receptor signaling pathway, NOD-like receptor signaling pathway, MAPK signaling pathway, IL-17 signaling pathway, and JAK–STAT signaling pathway. Additionally, several metabolites, such as arachidonic acid, L-lysine, 5Z, 8Z, 11Z, 14Z, 17Z-eicosapentaenoic acid, and 7,10,13,16-docosatetraenoic acid, showed differences between the challenged group and the immunization-challenged group. Previous studies have demonstrated the significant anti-inflammatory or antiviral effects of these metabolites (Asha and Sharma-Walia, 2021). Furthermore, our study identified differential expression of various metabolites, including indole, threonine, 3-hydroxycaprylic acid, tryptophan, α-dimorpholinic acid, essentalic acid, epinephrine, etc., between the PRV infection group and the immunization-challenged group. Moreover, we found that PRV can activate the PI3K/AKT/mTOR signaling pathway to regulate cell apoptosis and combat PRV-infected cells. Our findings suggest that several metabolites, such as histidine, L-tyrosine, glutathione, and dihydroartemisinin, have potential anti-PRV effects. Overall, this study confirms the potential of metabolites for therapeutic protection against PRV infection-induced acute encephalitis, providing valuable references and research directions for the treatment and prevention of PRV infection.

## Data availability statement

The original contributions presented in the study are included in the article/Supplementary material. The datasets presented in this article are not readily available because the project associated with this article is still ongoing. Requests to access the datasets should be directed to 601473235@qq.com.

## Ethics statement

The animal study was approved by the Ethics Committee of Sichuan Agricultural University. The study was conducted in accordance with the local legislation and institutional requirements.

## Author contributions

LX: Conceptualization, Data curation, Formal analysis, Software, Writing – original draft, Writing – review & editing. YZha: Formal analysis, Supervision, Writing – review & editing. QT: Formal analysis, Software, Writing – review & editing. TX: Formal analysis, Writing – review & editing. FL: Writing – original draft. LD: Software, Writing – original draft. ZJ: Supervision, Writing – review & editing. JZ: Supervision, Writing – review & editing. YY: Supervision, Writing – review & editing. SL: Funding acquisition, Project administration, Writing – review & editing. YZho: Resources, Writing – original draft. ZX: Conceptualization, Funding acquisition, Project administration, Resources, Validation, Writing – review & editing. LZ: Conceptualization, Funding acquisition, Project administration, Writing – original draft.
