# Supplementary material for: Transcriptome and metabolome analysis reveals PRV XJ delgE/gI/TK protects intracranially infected mice from death by regulating the inflammation
Source: Front Microbiol. 2024 Mar 14;15:1374646. doi: 10.3389/fmicb.2024.1374646 (PMC10972889; doi:10.3389/fmicb.2024.1374646)
Supplement: Supplementary file 1 [file Table_1.DOCX]

Supplementary Material

# Supplementary Figures


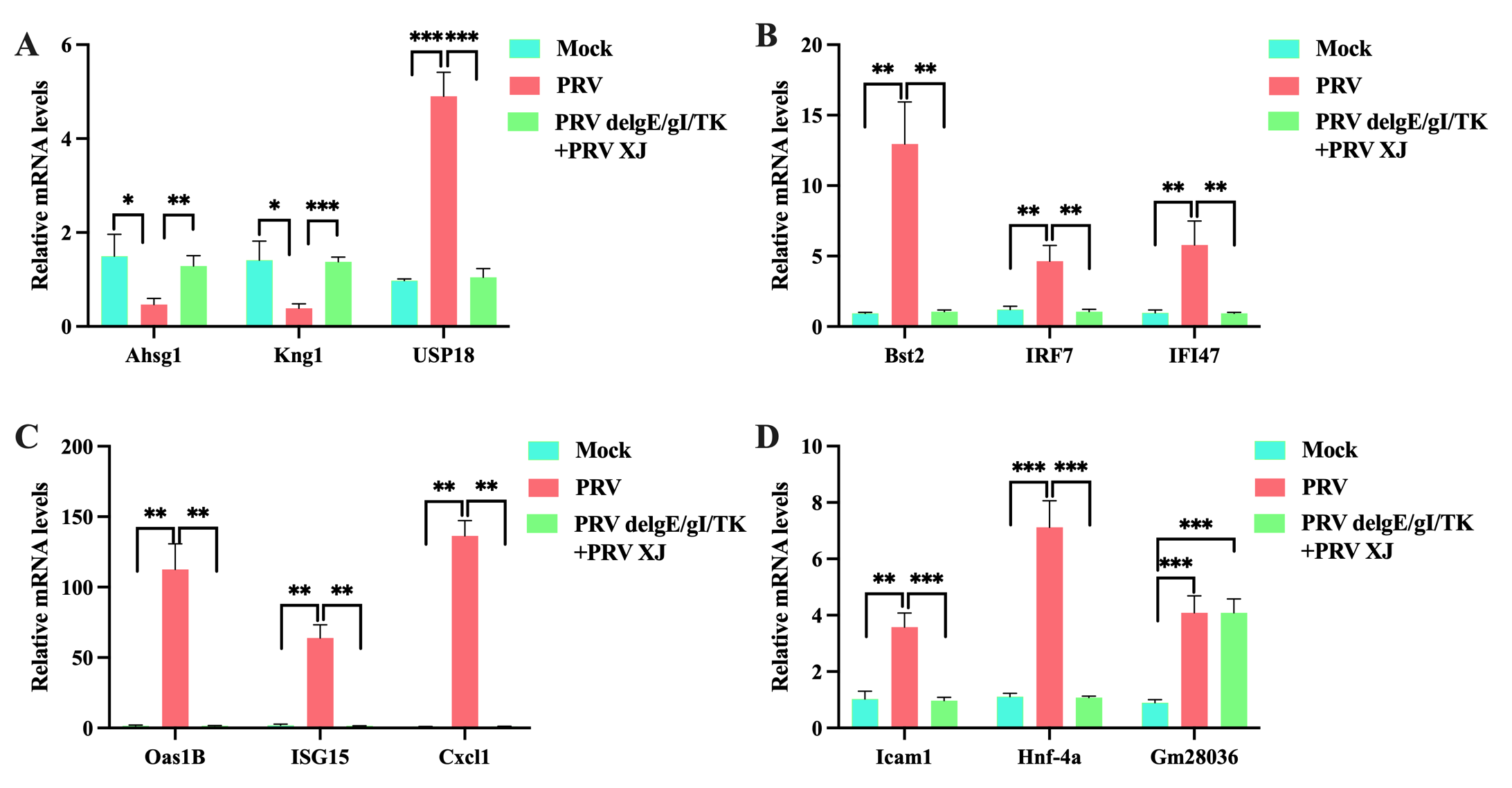


**Supplementary Figure 1.** Verification of the expression patterns of RNA-seq results using qRT-PCR. One-way ANOVA was used for analysis. *p<0.5, **p< 0.01, ***p< 0.001, ****p< 0.0001.


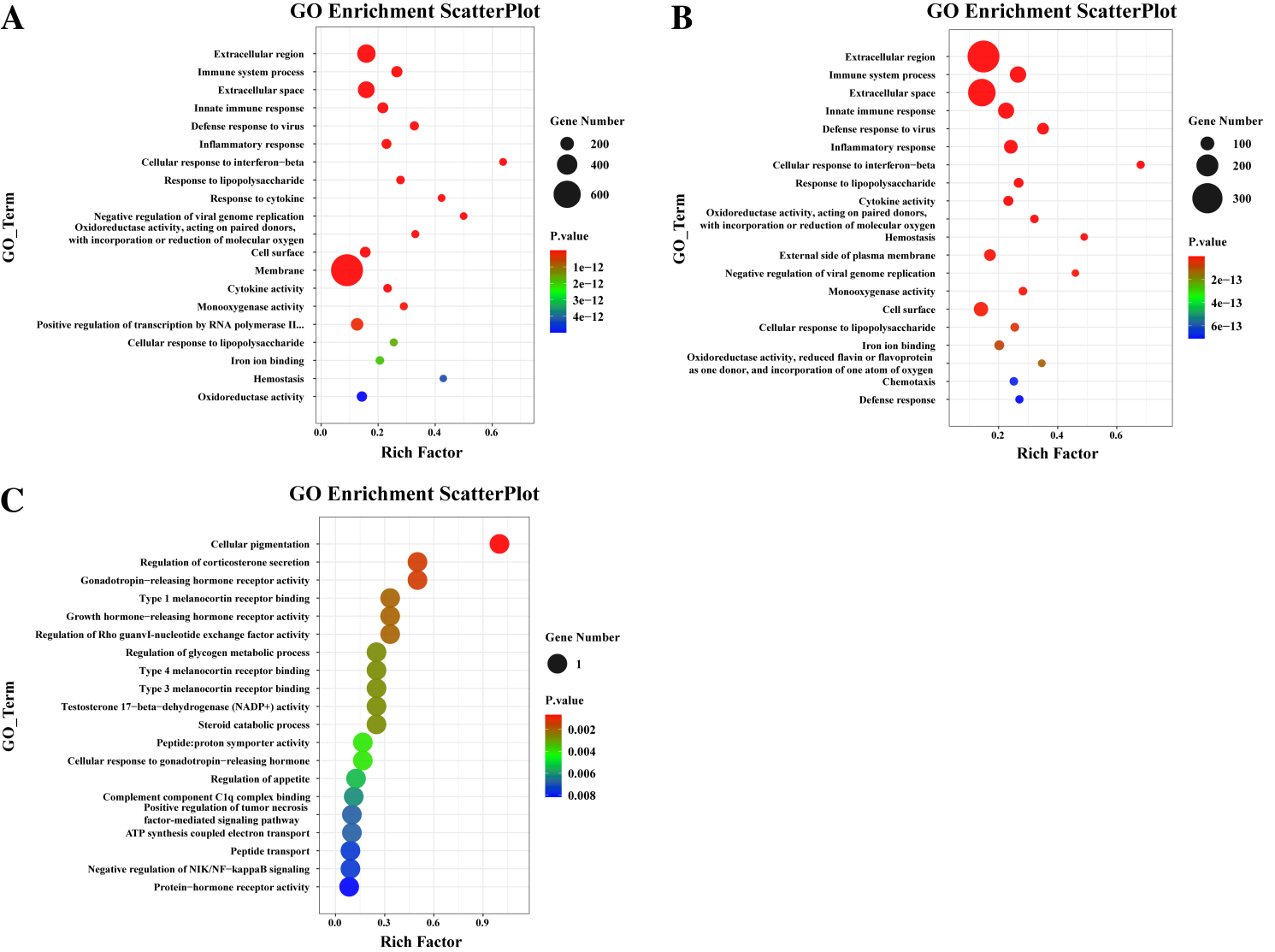


**Supplementary Figure 2.** The GO enrichment analysis of differential expression genes. (A) Scatterplot of the GO enrichment by differential expression genes for the immunization-challenged group versus the challenged group. (B) Scatterplot of the GO enrichment by differential expression genes for the challenged group versus the mock group. (C) Scatterplot of the GO enrichment by differential expression genes for the immunization-challenged group versus the mock group.
